# Long-term expansion of directly reprogrammed keratinocyte-like cells and in vitro reconstitution of human skin

**DOI:** 10.1186/s12929-020-00642-1

**Published:** 2020-04-20

**Authors:** Jie Zheng, Wonjin Yun, Junghyun Park, Phil Jun Kang, Gilju Lee, Gwonhwa Song, In Yong Kim, Seungkwon You

**Affiliations:** 1 Laboratory of Cell Function Regulation, Department of Biotechnology, College of Life Sciences and Biotechnology, Korea University, Seoul, 02841 Republic of Korea; 2 Institute of Animal Molecular Biotechnology, College of Life Sciences and Biotechnology, Korea University, Seoul, 02841 Republic of Korea; 3 Department of Pathology, College of Medicine, Korea University Guro Hospital, Seoul, 08308 Republic of Korea

**Keywords:** Induced keratinocyte-like cells, Urine cells, Direct lineage reprogramming, Long-term expansion, Skin reconstitution

## Abstract

**Background:**

Human keratinocytes and derived products are crucial for skin repair and regeneration. Despite substantial advances in engineered skin equivalents, their poor availability and immunorejection remain major challenges in skin grafting.

**Methods:**

Induced keratinocyte-like cells (iKCs) were directly reprogrammed from human urine cells by retroviral transduction of two lineage-specific transcription factors BMI1 and △NP63α (BN). Expression of keratinocyte stem cell or their differentiation markers were assessed by PCR, immunofluorescence and RNA-Sequencing. Regeneration capacity of iKCs were assessed by reconstitution of a human skin equivalent under air-interface condition.

**Results:**

BN-driven iKCs were similar to primary keratinocytes (pKCs) in terms of their morphology, protein expression, differentiation potential, and global gene expression. Moreover, BN-iKCs self-assembled to form stratified skin equivalents in vitro.

**Conclusions:**

This study demonstrated an approach to generate human iKCs that could be directly reprogrammed from human somatic cells and extensively expanded in serum- and feeder cell-free systems, which will facilitate their broad applicability in an efficient and patient-specific manner.

## Background

Major skin injuries and diseases caused by thermal burns, surgical incisions, infection, trauma, or chronic ulcers require medical intervention to heal properly and can be life-threatening in severe cases. In particular, burn injuries are often devastating and have long-term physical and psychosocial impacts. Such injuries can result in cosmetic disfigurement, meaning sufferers have to cope with an alteration in their appearance [1]. Current therapeutic protocols are based on debridement and application of skin grafts to protect the exposed layers and to allow reconstitution of the damaged portion. Autologous grafting allows full integration into the donor site; however, this approach is limited by the amount of skin available for wound coverage and rapid re-epithelialization as well as the creation of additional deficits, especially in cases with severe burns and skin morbidities [2, 3]. Allogeneic and xenogeneic skin grafts are also employed due to their better availability and accessibility of material for wound closure. However, their clinical use is limited by the recipient’s immune response, which leads to cellular destruction, and therefore these grafts are only used as temporary biologic dressings [4]. Consequently, much attention has been paid to generating an unlimited and immunocompatible graft material that provides therapeutic benefit as a skin replacement. One approach is to use autologous keratinocytes that have been expanded in vitro for 2–4 weeks [5, 6]. Proof-of-principle studies demonstrated that these cells induce rapid re-epithelialization; however, the functional and aesthetic outcomes, clinical feasibility, and cost-benefit relationship of remote culture facilities remain unclear [7, 8].

Stem cells and derived products are potential therapeutics in dermatology and reconstructive surgery. Substantial advances in differentiating pluripotent stem cells, including embryonic stem cells (ESCs) and induced pluripotent stem cells (iPSCs), mean that patient- and disease-specific stem cells can be obtained, which will help to improve understanding of human development and disease processes, develop new therapeutics, and pave the road to personalized medicine [9]. However, these approaches carry the risks of tumor formation and immune rejection, and their application is hampered by ethical concerns [9, 10]. Alternatively, reprogramming of somatic donor cells, bypassing an intermediate pluripotent stage, provides important benefits including karyotypic stability, homogeneity of the target cell population, low tumorigenic risk, patient specificity, and time- and labor-efficient processing [11]. Intensive efforts to manipulate cell fates employing a set of lineage-specific transcription factors and small molecules recently led to the generation of various cell types, including adult stem/progenitor cells (oligodendrocyte progenitor cells, hematopoietic progenitor cells, neural stem cells, and limbal stem cells) and somatic cells (neurons, cardiomyocytes, hepatocytes, and pancreatic β cells) [11]. Despite these impressive exhibitions of phenotypic conversion, only one study reported transcription factor-driven transdifferentiation of human somatic cells into skin keratinocyte lineage cells, to the best of our knowledge. Chen et al. reported that two keratinocyte-specific transcription factors, called △NP63α and KLF4 (NK), can directly convert human neonatal dermal fibroblasts and human colon carcinoma cells into cells with a basal keratinocyte phenotype [12]. These keratinocyte-like cells (KCs) resemble primary keratinocytes (pKCs) in terms of their morphology, biological characteristics, and global gene expression, but fail to form a stratified epidermis.

Epidermal stem/progenitor populations, found in epidermis with high proliferative capacity, and differentiated keratinocytes are distributed in tightly adherent layers of the epidermis and are organized into distinct zones according to their differentiation stages. Differentiating keratinocytes progressively emerge from the proliferative basal layer and replenish the upper layer by undergoing renewal throughout life, thereby desquamating the dead envelope at the skin surface [13]. In the basal layer of the epidermis, keratinocytes not only maintain tissue homeostasis, but also participate in skin repair following injury [14, 15]. However, the restricted in vitro proliferative potential of isolated epidermal populations, including keratinocyte stem/progenitor cells, hinders their preclinical and clinical applications. Cultured keratinocytes were reported to irreversibly lose their stem cell properties upon passage [16, 17]. Using isolated murine keratinocyte subpopulations, Redvers et al. and Blanpain et al. showed that the expansion and functional properties of keratinocytes are only retained in a specific environment [18, 19]. Long-term expandability of keratinocytes is an indispensable prerequisite for potential medical applications, including cell therapy, disease modeling, drug screening, and reconstitution of functional skin in vitro.

Here, we established induced KC (iKC) lines from human urine-derived cells, which were obtained from two healthy donors (one male and one female), by forced expression of two transcription factors called BMI1 and △NP63α (BN) under keratinocyte culture conditions. Urine-derived cells provide the following advantages: i) these cells are available regardless of a person’s age, gender, or health condition; ii) these cells can be collected by a simple, safe, inexpensive, and non-invasive procedure; and iii) primary cells can be easily isolated without enzymatic digestion [20]. BN-driven iKCs (BN-iKCs) closely resembled pKCs in terms of their morphology, biological characteristics, in vitro differentiation capacities, and global gene expression. More importantly, BN-iKCs stably expanded in serum- and feeder cell-free conditions and self-assembled to form stratified skin equivalents in vitro.

## Methods

### Cell culture

pKCs (human neonatal epidermal keratinocytes; Thermo Fisher Scientific, Waltham, MA, USA) were cultured in a Matrigel (Corning, New York, NY, USA)-coated plate in keratinocyte serum-free medium (KSFM). This medium was a 1:1 ratio of Defined Keratinocyte-SFM (Thermo Fisher Scientific):Keratinocyte-SFM (Thermo Fisher Scientific) containing 1% penicillin/streptomycin (P/S; Lonza, Basel, Switzerland), 2 mM L-glutamine (Lonza), 10 μM Y-27632 (Tocris, Bristol, UK), and 1 μM A-83-01 (Tocris). When pKCs reached ~ 80% confluency, they were subcultured in ReagentPack™ Subculture Reagents (Lonza) in a Matrigel (Corning)-coated plate. Mouse 3 T3-J2 cells (Kerafast, Boston, MA, USA) were maintained in high-glucose DMEM (Hyclone, Logan, UT, USA) supplemented with 10% fetal calf serum (Thermo Fisher Scientific), 1% P/S, and 2 mM L-glutamine.

### Isolation and culture of urine cells

Urine samples were obtained from two healthy donors (one male and one female). Cells were isolated from ~ 1000 ml of urine by modifying a previously described method [21]. Briefly, cells in urine samples were collected by centrifugation and seeded in a gelatin (Sigma, St. Louis, MO, USA)-coated 12-well plate in urine primary culture medium, and then 500 μl of fresh medium was added every day. After 4–5 days, the medium was replaced by urine proliferation medium for further culture of attached urine cells (regarded as passage 1). Urine primary culture medium was DMEM/F12 (Hyclone) containing 10% fetal bovine serum (FBS; Hyclone), 1% penicillin/streptomycin/fungizone solution (Hyclone), and 2 mM L-glutamine. Urine proliferation medium was a 1:1 ratio of high-glucose DMEM:REGM (Lonza) containing 5% FBS, 0.5% P/S, 2 mM L-glutamine, 1% non-essential amino acid solution (Thermo Fisher Scientific), 2.5 ng/ml basic fibroblast growth factor (bFGF; PeproTech, London, UK), and 2.5 ng/ml epidermal growth factor (EGF, PeproTech).

### Construction of retroviral plasmids

The human BMI1 fragment was excised from the pBabe-hBMI1 vector (provided by Goberdhan P. Dimri) using the restriction enzyme EcoRI (Takara Bio Inc., Kusatsu, Japan) and inserted into the EcoRI site of the pMXs-puro backbone vector (Cell Biolabs Inc., San Diego, CA, USA). The human △NP63α fragment was excised from the deltaNp63alpha-FLAG expression vector (plasmid #26979; Addgene, Watertown, NY, USA) using the restriction enzymes BamHI (Takara Bio Inc.) and NotI (Takara Bio Inc.), and inserted into the BamHI and NotI site of the pMXs-puro backbone vector. The constructed plasmids were confirmed by sequencing.

### Generation of induced KCs

Retroviruses were produced by transfecting the PT67 amphotropic packaging cell line (Clontech Laboratories, Mountain View, CA, USA) with pMXs-based retroviral vectors encoding human BMI1, △NP63α, and KLF4 (plasmid #17219, Addgene) using a lipofectamine 2000-based protocol (Thermo Fisher Scientific). Retrovirus-containing supernatants were harvested at 72–120 h post-transfection, filtered with a 0.45 μm sterile syringe filter (Millipore, Burlington, VT, USA), and concentrated by ultracentrifugation (20,000 g, 1.5 h, 4 °C).

Urine cells at passage 3 were infected with the resulting retroviruses, either alone or in combination. After 2 days, infected urine cells were re-seeded at a density of 2 × 10^4^ cells per well in a 12-well plate with a mitomycin-inactivated 3 T3-J2 feeder layer, at an initial seeding density of 2 × 10^4^ cells/cm^2^, in keratinocyte growth medium (KGM) containing 2% FBS (2% FKGM), which was refreshed every 2 days for ~ 2 weeks. Formed keratinocyte colonies were picked and re-plated as individual clones in a type I collagen-coated 24-well plate in KGM containing 10% FBS (10% FKGM). A picked single colony was further selected and expanded in 10% FKGM. FKGM was a 3:1 ratio of DMEM/F12 containing 2% or 10% FBS, 1% P/S, 2 mM L-glutamine, 5 μg/ml insulin (Sigma), 0.5 μg/ml hydrocortisone (Sigma), 8.3 ng/ml cholera toxin (Sigma), 1.37 ng/ml triiodothyronine (Sigma), 52.8 μg/ml ascorbic acid 2-phosphate (Sigma), 20 ng/ml EGF, and 10 μg/ml adenine (Sigma) [22]. To generate iKCs in serum-free conditions, BN-infected urine cells at passage 3 or human foreskin fibroblasts (CRL-2522, ATCC, Manassas, VA, USA) at passage 5 were seeded at a density of 2 × 10^4^ cells per well in a 12-well plate with a mitomycin-inactivated 3 T3-J2 feeder layer in 2% FKGM and cultured for ~ 5–6 days. Thereafter, the medium was replaced by KSFM and cells were cultured for a further 7–8 days. Single formed colonies were picked, selected, and propagated in a type I collagen-coated plate in KSFM.

### Immunofluorescence

Cultured cells were fixed in 4% paraformaldehyde solution (Biosesang, Seongnam, South Korea) for 20 min at room temperature and washed three times with 1× DPBS (Welgene Inc., Gyeongsan, South Korea). Permeabilization and blocking were performed with 1× DPBS containing 5% donkey serum (Millipore) and 0.3% Triton X-100 (Sigma) for 30 min at room temperature to detect nuclear/cytoplasmic proteins or with 1× DPBS containing 5% donkey serum and 0.1% Triton X-100 for 10 min at room temperature to detect surface proteins. Thereafter, cells were washed three times and incubated with primary antibodies diluted in 1× DPBS containing 5% donkey serum at 4 °C overnight. After incubation, cells were washed three times and incubated with secondary antibodies diluted in 1× DPBS at room temperature for 60 min. Nuclei were stained with DAPI (Sigma). The antibodies are listed in Supplementary Table S1.

### RT-PCR and real-time PCR

Total RNAs were isolated from cells using TRIzol (Thermo Fisher Scientific), and cDNAs were synthesized using RT PreMix (Bioneer, Daejeon, South Korea) and the oligo dT primer (Bioneer) according to the manufacturers’ instruction. Gene fragments were amplified using 25 ng of cDNA, specific primers (Bioneer), and PCR PreMix (Bioneer) in a total reaction volume of 20 μl. All RT-PCR amplifications were verified to be in the linear range. Real-time PCR was performed using a CFX Connect™ Real-Time PCR Detection System (Bio-Rad, Hercules, CA, USA) and SYBR Green Supermix (Bio-Rad). The housekeeping gene GAPDH was used as an internal standard. The primer sequences are listed in Supplementary Table S2.

### Colony-forming assay

Cells were seeded at a density of 2.5 × 10^3^ cells per well in a 6-well plate with a mitomycin-inactivated 3 T3-J2 feeder layer and cultured in 10% FKGM or KSFM for 7 days. Thereafter, cells were fixed in 10% formalin for 20 min at room temperature and stained with 0.1% crystal violet solution for 20 min at room temperature.

### Differentiation of pKCs and iKCs

To generate terminally differentiated keratinocytes, pKCs (passage 2) or BN-iKCs (passage 6) were seeded in a collagen-coated 6-well plate and maintained until they reached 80–90% confluency. Thereafter, cells were differentiated in Keratinocyte-SFM containing 1.2 mM CaCl_2_ for 5 days and 100 nM **Phorbol 12-myristate 13-acetate (PMA, Sigma) for 2 days**. Differentiation of sebocytes was performed as previously described [23]. pKCs (passage 2) or BN-iKCs (passage 6) were seeded at a density of 2 × 10^5^ cells per well in a 6-well plate in sebocyte induction medium, which was DMEM/F12 containing 6% FBS, 2% human serum (Sigma), 1% P/S, and 10 ng/ml EGF, and cultured overnight. Thereafter, the medium was replaced by sebocyte induction medium containing 10 nM insulin, 1 μM troglitazone (Cayman, Ann Arbor, MI, USA), 100 μM WY14643 (Sigma), and 10 nM DMSO (Sigma) to induce sebocyte differentiation, and cells were cultured for a further 12 days. Differentiated cells were washed twice with 1× DPBS and fixed with 10% formalin for 30 min at room temperature. Fixed cells were washed and pretreated with 60% isopropanol for 2 min and incubated with 0.5% (w/v) Oil red O solution for 30 min at room temperature. Stained cells were rinsed with 60% isopropanol and imaged under a phase-contrast microscope (Olympus, Tokyo, Japan).

### RNA-sequencing and analysis

Total RNA was isolated using TRIzol. RNA quality was assessed using an RNA 6000 Nano Chip and Agilent 2100 bioanalyzer (Agilent Technologies, Santa Clara, CA, USA). Libraries were prepared using ~ 2 μg of total RNA and a SMARTer Stranded RNA-Seq Kit (Clontech Laboratories). mRNA was isolated from total RNA using a Poly(A) RNA Selection Kit (Lexogen, Greenland, AR, USA) in accordance with the manufacturer’s instructions and used for cDNA synthesis and shearing. Indexing was performed using Illumina indexes 1–12. The enrichment step was carried out using PCR. Subsequently, libraries were checked using the Agilent 2100 bioanalyzer with a DNA High Sensitivity Kit to evaluate the mean fragment size. Quantification was performed on a StepOne Real-Time system (Thermo Fisher Scientific) using a Library Quantification Kit. High-throughput paired-end 100 bp sequencing was performed using a HiSeq 2500 system (Illumina, San Diego, CA, USA).

The alignment files were obtained from mapped mRNA-seq reads using TopHat software [24]. Differentially expressed genes were determined based on counts from unique and multiple alignments using coverage in Bedtools [25]. RT (read count) data were processed based on the quantile normalization method using EdgeR within R (R Development Core Team, 2016) and Bioconductor [26]. The alignment files were also used to assemble transcripts, estimate their abundances, and detect differential expression of genes or isoforms using cufflinks or DESeq2 (FDR adjusted *P* < 0.05). Fragments per kilobase of exon per million fragments (FPKM) was calculated to determine the expression levels of the gene regions. Gene classification was based on searches performed with DAVID (http://david.abcc.ncifcrf.gov/).

### In vitro skin reconstitution

Skin equivalents were generated using an air-liquid interface method as previously reported [27, 28]. Foreskin fibroblasts (CRL-2522; ATCC, Manassas, VA, USA) were encapsulated in a rat tail type I collagen gel (Corning), plated into a 6-well cell culture insert (3.0 mm polycarbonate membrane, Corning) at a density of 2 × 10^5^ cells per well to form an acellular layer, and cultured in fibroblast medium for ~ 1 week. pKCs at passage 2, BN-MiKCs at passage 6, and male donor-derived urine cells (M-UCs) at passage 3 were seeded onto the formed acellular layer at a density of 3 × 10^5^ cells per well and cultured in keratinocyte differentiation medium 1 for 1 week, during which time the medium was replaced with fresh medium every 2 days. Thereafter, the surface of cultured cells was exposed to air for air-liquid interface culture, keratinocyte differentiation medium 1 was replaced every 2 days for 1 week, and then keratinocyte differentiation medium 2 was replaced every 2 days for an additional 1 week. Fibroblast medium was high-glucose DMEM containing 10% FBS, 1% P/S, 2 mM L-glutamine, 4 ng/ml bFGF (PeproTech), and 50 μg/ml ascorbic acid 2-phosphate. Keratinocyte differentiation medium 1 was a 1:1 ratio of DMEM/F12 containing 1.39 mg/ml NaHCO_3_, 1% P/S, 2 mM L-glutamine, 0.4 μg/ml hydrocortisone, 5 μg/ml insulin, 5 μg/ml transferrin (Sigma), 0.02 nM triiodothyronine, 0.18 mM adenine, 0.1 mM ethanolamine (Sigma), 0.1 mM phosphorylethanolamine (Sigma), 0.0053 nM selenium (Sigma), 50 μg/ml gentamycin (Lonza), 1.2 mM CaCl_2_, and 2% FBS. Keratinocyte differentiation medium 2 was keratinocyte differentiation medium 1 containing 1% FBS instead of 2% FBS.

### Immunohistochemistry

Samples cultured at the air-liquid interface were fixed in 4% paraformaldehyde at 4 °C, embedded in paraffin, and dissected into 4 μm thick sections. Sections were stained with hematoxylin and eosin (H&E, Sigma) for routine histological evaluation. For immunohistochemistry, sections were deparaffinized with xylene, and antigen recovery was performed by boiling in citrate buffer (pH 6.0) for 10 min. Next, the sections were stained with specific antibodies against KRT14, Loricrin, and Involucrin using a *Vectastain ABC Kit* (Vector Laboratories, Burlingame, CA, USA). Nuclei were counterstained with hematoxylin (Sigma). The antibodies are listed in Supplementary Table S1.

### Statistical analysis

Data are expressed as mean values ± SD in n independent observations. Data were compared using a one-way ANOVA and the paired two-tailed Student’s t test. *P* < 0.05, *P* < 0.01, or *P* < 0.001 was considered statistically significant.

## Results

### Generation of iKCs from human urine cells

**Fig. 1:**
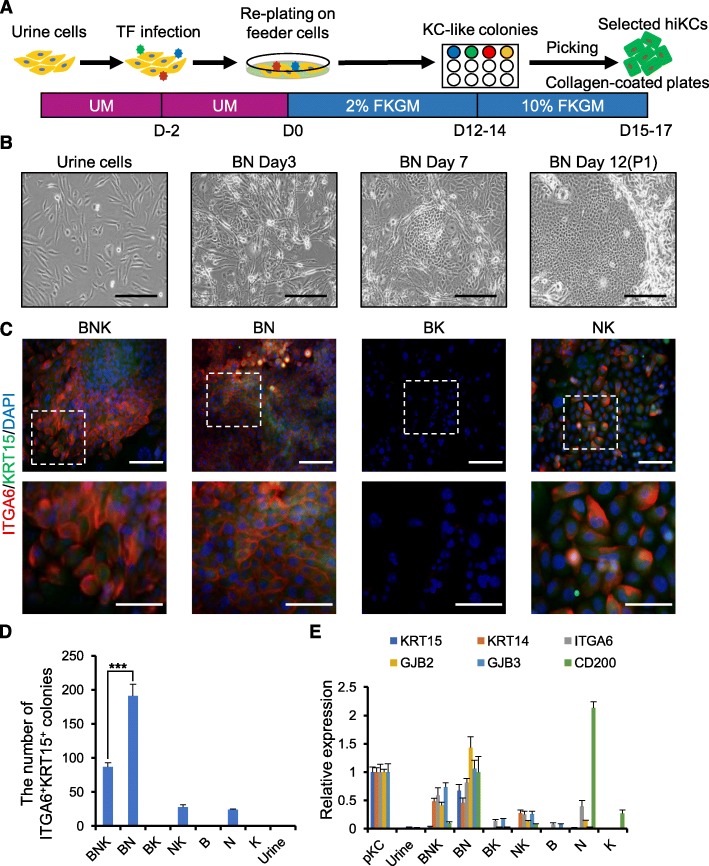
Direct reprogramming of human urine cells into iKCs. **a** Schematic diagram of the induction of iKCs from human urine cells. Transcription factors (TFs). **b** Morphological changes of urine cells during direct reprogramming. At passage 3, urine cells were infected with retroviruses encoding BN in urine proliferation medium. BN-infected urine cells were plated in 2% FKGM on a 3 T3-J2 feeder layer to form holoclones. At day 12, the holoclones were picked for further expansion and characterization. Scale bars = 500 μm. **c** Immunostaining of the stem cell markers KRT15 and ITGA6 in BNK-, BN-, BK-, and NK-infected cells with specific antibodies at day 12 post-induction. Nuclei were counterstained with DAPI. The lower row shows magnified images of the boxed areas in the upper row. Scale bars = 200 μm (upper), 100 μm (lower). **d** Number of ITGA6^+^KRT15^+^ colonies formed by BNK-, BN-, BK-, NK-, B-, N-, and K-infected cells and urine cells at 12 days post-induction. Cells were seeded at a density of 2 × 10^4^ cells per well in a 12-well plate. Data represent the mean ± SEM. *** *P* < 0.001. **e** qRT-PCR analysis of relative expression of stem cell markers in BNK-, BN-, BK-, NK-, B-, N-, and K-infected cells, urine cells, and pKCs at 12 days post-induction. Data represent the mean ± SEM. GAPDH was used as a loading control. B, BMI1; N, △NP63α; and K, KLF4

Urine samples contain heterogeneous cell populations and adherent cells removed from the renal tubules or urethras [29, 30]. Due to their good accessibility and high availability, human urine cells are considered to be a promising source of material for cellular reprogramming and personalized cell therapies [20]. Previous studies showed that urine cells isolated from the same donor exhibit two different types of cobblestone-like (Type I) and elongated (Type II) morphology during isolation, and the latter cells possessed a higher proliferative potential and reprogramming efficiency than the former cells [21, 29]. Accordingly, Type II urine cells were chosen for this study. Prior to directly reprogramming urine cells into iKCs, we investigated expression of several epidermal keratinocyte lineage markers (KRT15, KRT14, ITGA6, KRT10, and Involucrin) in urine cells. None of these markers were expressed (Figure. S1A). Based on a previous report of NK-driven conversion of human neonatal foreskin fibroblasts into iKCs [12], we first infected human urine cells, with retroviruses encoding NK and cultured them in 2% FKGM with 3 T3-J2 feeder cells (Fig. 1a, S2A). NK-overexpressing urine cells exhibited a colony morphology and expressed keratinocyte stem cell markers (Fig. 1c–e and S2A); however, these cells failed to expand in 10% FKGM for more than three passages (Figure. S3E). Considering that KLF4 is highly expressed during induction into terminal differentiated keratinocytes [31, 32] and △NP63α-triggered epithelial-mesenchymal transition of normal primary human epidermal keratinocytes [33], we hypothesized that BMI1, rather than KLF4, would improve reprogramming of urine cells into iKCs and acquisition of epidermal stemness. BMI1, a stem cell factor in neural and hematopoietic stem cells [34, 35], is detected in epidermal basal/suprabasal layers, and its ectopic expression contributes to survival and proliferation of keratinocytes and reversal of △NP63α-triggered epithelial-mesenchymal transition by inhibiting the transforming growth factor β (TGFβ) signaling pathway [33, 36, 37]. Accordingly, urine cells were infected with retroviral vectors encoding BMI1, △NP63α, and KLF4 either alone or in combination (B, N, K, BN, BK, NK, and BNK). Putative iKCs, which exhibited a holoclone morphology similar to that of expandable keratinocytes, were observed upon infection with BNK or BN. In the adult human skin, it has been reported that CD71^dim^, ITGA6^Bri^ and KRT15 are more dominant in deep rete ridges where stem and transient amplifying cells are abundant, suggesting that KRT15 and ITGA6 could serve as a specific marker for identification of keratinocyte stem and transient amplifying epidermal cells [38]. A high percentage of the colonies expressed KRT15 and ITGA6, whereas these markers were not expressed in non-infected urine cells and feeder cells (Fig. 1a–c, S1, and S2). BN-overexpressing urine cells had the highest KRT15^+^ITGA6^+^ colony-forming efficiency (CFE), and these colonies expressed stem cell markers at a comparable level as pKCs (Fig. 1d, e). These findings indicate that urine cells are efficiently converted into keratinocyte-like colonies via overexpression of BN.

### Selection and long-term culture of BN-iKCs

**Fig. 2:**
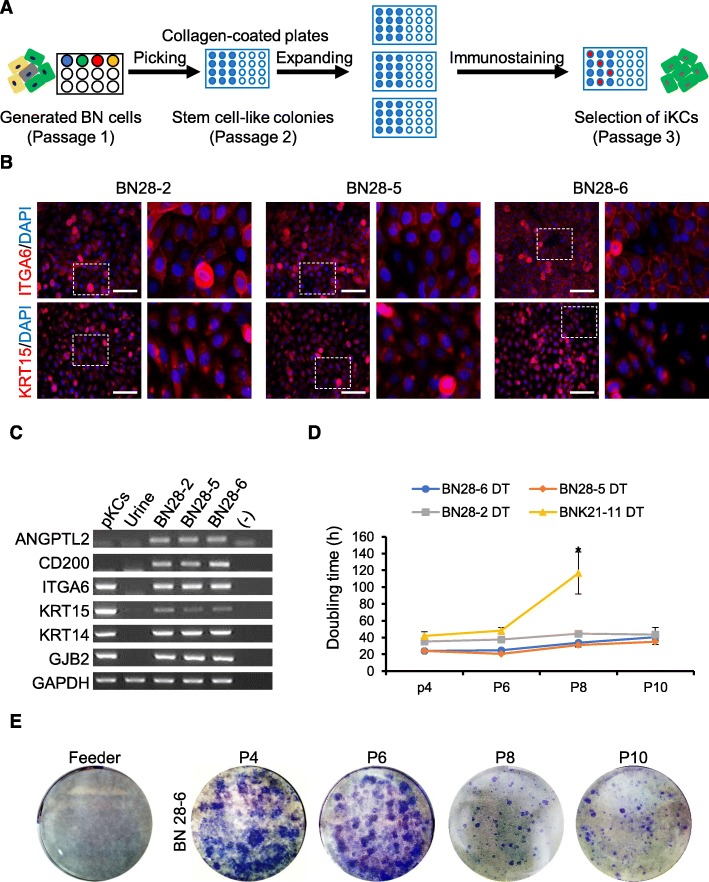
Selection, characterization, and expansion of BN-iKCs. **a** Schematic diagram of the selection of ITGA6^+^KRT15^+^ iKCs. **b** Immunostaining of selected ITGA6^+^KRT15^+^ BN-iKCs (BN28–2, BN28–5, and BN28–6) at passage 3 with specific antibodies against ITGA6 and KRT15. Nuclei were counterstained with DAPI. Images on the right are magnified views of the boxed areas in images on the left. Scale bars = 200 μm. **c** RT-PCR analysis of stem cell marker expression in pKCs, urine cells, and BN-iKCs. **d** Doubling time of BN-iKCs and BNK21–11 cells during long-term expansion. Cells were seeded at a density of 5 × 10^4^ cells per well in a 6-well plate. Data represent the mean ± SEM. * *P* < 0.05. **e** Colony-forming assay of BN-iKCs during long-term expansion. BN-iKCs were seeded at a density of 0.25 × 10^4^ cells per well in a 6-well plate with a 3 T3-J2 feeder layer and cultured for 1 week. The plates were stained with crystal violet

A homogeneous population of BN-iKCs was attained by single-colony picking and KRT15/ITGA6 immunostaining for further expansion and maintenance (Fig. 2a). We selected three KRT15^+^ITGA6^+^ colonies from putative BN-iKCs (referred to as line BN28–2, BN28–5, and BN28–6) and one KRT15^+^/ITGA6^+^ colony from each of putative BNK-, NK-, and N-iKCs (referred to as line BNK21–11, NK16–6, and N20–10, respectively) (Fig. 2b and S3A–D). BN-iKCs co-expressed KRT15 and ITGA6; however, KRT15 expression was lower in BNK21–11, NK16–6, and N20–10 cells (Fig. 2b and S3A–C). Intriguingly, BN-iKCs not only expressed normal stem cell markers (KRT15, KRT4, ITGA6, and GJB2), but also CD200 and ANGPTL2, which are specifically expressed in human hair follicle epithelial stem cells (HFSCs) (Fig. 2c). Meanwhile, the current standard method for long-term expansion of human keratinocytes, which was pioneered by Rheinwald and Green [39], uses a feeder layer of 3 T3-J2 mouse fibroblasts, which are irradiated to prevent their proliferation and to suppress differentiation of keratinocytes. Importantly, the presence of other cell types or serum carries the risks of viral infection and pathogen transmission in a clinical setting and affects the morphology and biology of target cells, which complicates the interpretation of research results [40, 41]. Therefore, we attempted to expand KRT15^+^ITGA6^+^ BN-iKCs on a collagen-coated plate in feeder cell-free conditions [42, 43]. The proliferation rate of BNK21–11 cells was higher than those of NK16–6 and N20–10 cells and further their population doubling time significantly increased in comparison with that of BN-iKCs at passage 8 (> 110 h), leading to growth arrest at the next passage (Fig. 2d). In addition, the colony-forming assay showed that BN-iKC colonies formed in the first six passages and then their size and number gradually decreased, losing of the characteristic of stemness, while BNK21–11 cells did not form colonies (Fig. 2e and S3F). NK16–6 and N20–10 cells could not be lasted more than 3 passages and exhibited senescence-like morphological changes and increased apoptosis (Figure. S3E). By contrast, the population doubling time of BN-iKCs was ~ 20–40 h and remained stable for at least 10 passages (Fig. 2d). These results suggest that BN-iKCs can undergo long-term expansion in feeder cell-free culture systems, while maintaining expression of stem cell markers and stemness properties.

### In vitro differentiation potential of BN-iKCs

**Fig. 3:**
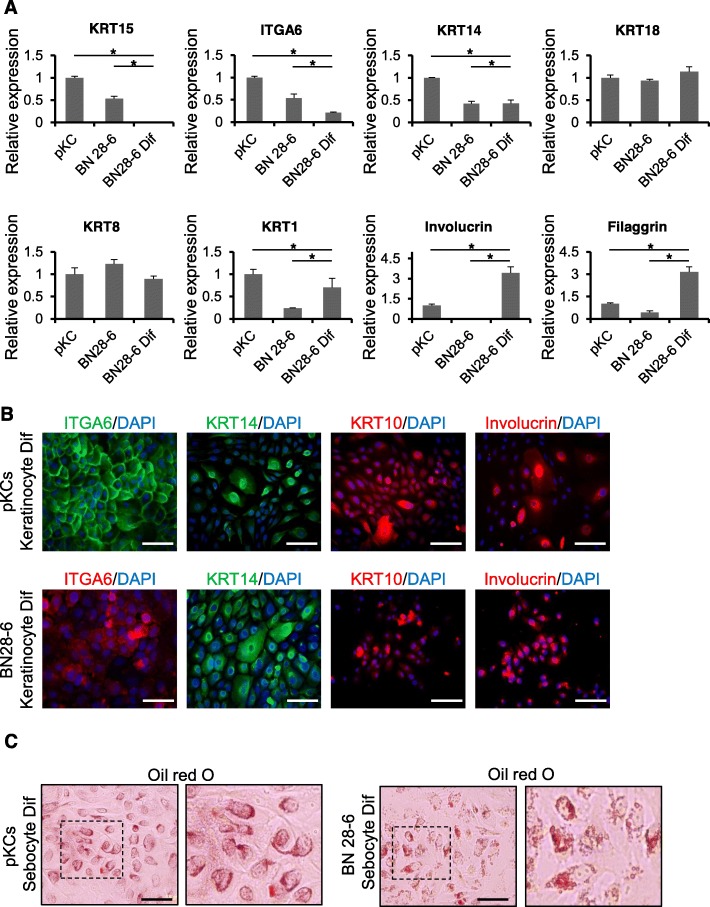
Differentiation potential of BN-iKCs in vitro. **a** Relative mRNA expression of keratinocyte lineage markers in pKCs, BN28–6 cells, and mature keratinocytes differentiated from BN28–6 cells. Stem cell markers: KRT15 and ITGA6; basal/suprabasal markers: KRT14, KRT8, and KRT18; differentiated markers: KRT1, Involucrin, and Filaggrin. GAPDH was used as a loading control. Data represent the mean ± SEM. * *P* < 0.05. **b** Immunofluorescence analysis of keratinocyte markers (ITGA6, KRT14, KRT10, and Involucrin) in mature keratinocytes differentiated from pKCs and BN28–6 cells. Nuclei were counterstained with DAPI. Scale bars = 200 μm. **c** Oil red O staining of sebocytes differentiated from pKCs and BN28–6 cells. Images on the right are magnified views of the boxed areas in images on the left. Scale bars = 200 μm

In vitro exposure to a high concentration of extracellular calcium induces differentiation of undifferentiated keratinocytes into mature keratinocytes [44]. The capacity of BN-iKCs to differentiate into keratinocytes was assessed by qPCR analysis and immunostaining. Expression of differentiated keratinocyte markers (KRT1/KRT10, Involucrin, and Filaggrin) was increased in differentiated BN-iKCs, while expression of stem cell markers (KRT15 and ITGA6) was concurrently decreased (Fig. 3a, b and S1C). KRT1 and KRT10 are a pair of keratins specifically expressed in suprabasal cells. In addition, expression of genes (KRT14, KRT8, and KRT18) that are enriched in both basal and differentiated keratinocytes did not significantly differ between undifferentiated and terminally differentiated BN-iKCs (Fig. 3a, b). Neither stem cell nor mature keratinocyte markers were expressed in urine cells cultured in differentiation conditions (Figure. S4). Abundant oil droplets were detected in the cytoplasm of BN-iKC- and pKC-derived sebocytes by Oil red O staining (Fig. 3c and S5), while no oil droplets were observed in urine cells (Figure. S5E, F). Furthermore, the reproducibility of this protocol was repeatedly evaluated utilizing female donor-derived urine cells (F-UCs). BN-transduced F-UCs formed keratinocyte-like colonies (Figure. S6A), expressed stem cell markers at the mRNA and protein levels (Figure. S6B, C), and had the potential to differentiate into mature keratinocytes (Figure. S6D). These results suggest that BN amplification is essential to establish KC identity in human urine cells.

### Generation and long-term expansion of BN-iKCs in serum-free culture conditions

**Fig. 4:**
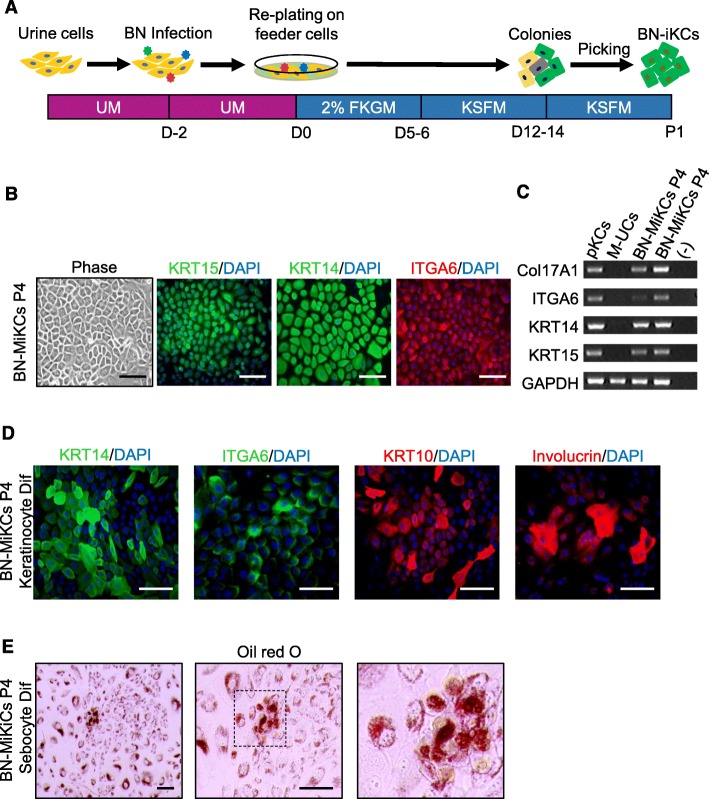
BN-driven direct reprogramming of M-UCs into iKCs in serum-free conditions. **a** Schematic diagram of the generation of BN-iKCs. **b** Phase-contrast image and immunostaining of stem cell markers (KRT15, KRT14, and ITGA6) in BN-MiKCs at passage 4 selected by single-colony picking. Nuclei were counterstained with DAPI. Scale bars = 200 μm. **c** RT-PCR analysis of stem cell markers (Col17A1, ITGA6, KRT14, and KRT15) in pKCs, M-UCs, and BN-MiKCs. **d** Expression of keratinocyte markers (ITGA6, KRT14, KRT10, and Involucrin) in mature keratinocytes differentiated from BN-MiKCs. Scale bars = 200 μm. **e** Oil red O staining of sebocytes differentiated from BN-MiKCs. The image on the right is a magnified view of the boxed area in the central image. Scale bars = 200 μm

FBS is an undefined and highly complex mixture containing various macromolecules, hormones, growth factors, lipids, and proteins. Variability in FBS in terms of donor, country, collection method, and processing unpredictably influence cultured cells [45]. For example, the presence of fetuin, high-density lipoprotein, platelet-derived growth factor, or TGFβ reportedly contributes to terminal differentiation of mouse epidermal keratinocytes and a reduction in CFE [46]. Similarly, the CFE of BN-iKCs decreased upon long-term serial passage in FKGM, leading us to speculate that FBS reduces the CFE. We therefore investigated whether the present protocol for long-term expansion and maintenance of BN-iKCs could be performed in KSFM (Fig. 4a). Initially, 2% FKGM was used to stably culture and transduce M- or F-UCs with BN, and then replaced by KSFM for further processing. After 12–14 days, keratinocyte-like colonies were collected and expanded in KSFM in type I collagen-coated plates. The selected colonies of BN-iKCs induced from M- or F-UCs (referred to as BN-MiKCs and BN-FiKCs, respectively) expressed exogenous BMI1 and △NP63α (Figure S7A, B). At passage 4, Both BN-MiKCs and BN-FiKCsexhibited a keratinocyte-like morphology and expressed stem cell markers (Fig. 4b, c, and S8A–C). Both BN-MiKCs and BN-FiKCs had the potential to differentiate into mature keratinocytes (Fig. 4d) and sebocytes (Fig. 4e and S9C). The expression of exogenous △NP63α showed a decrease in both BN-MiKCs and BN-FiKCs during terminal differentiation of keratinocytes (Figure S7C). Furthermore, we investigated the response of BN-iKCs to a PKC activator, PMA, known as keratinocyte terminal differentiation inducer [47]. Consistent with previous study, exposure of BN-MiKCs and BN-FiKCs to PMA allowed the expression of terminal differentiation markers (Involucrin and Loricrin) and blocked the expression of early differentiation marker (KRT10) (Figure S7D).

**Fig. 5:**
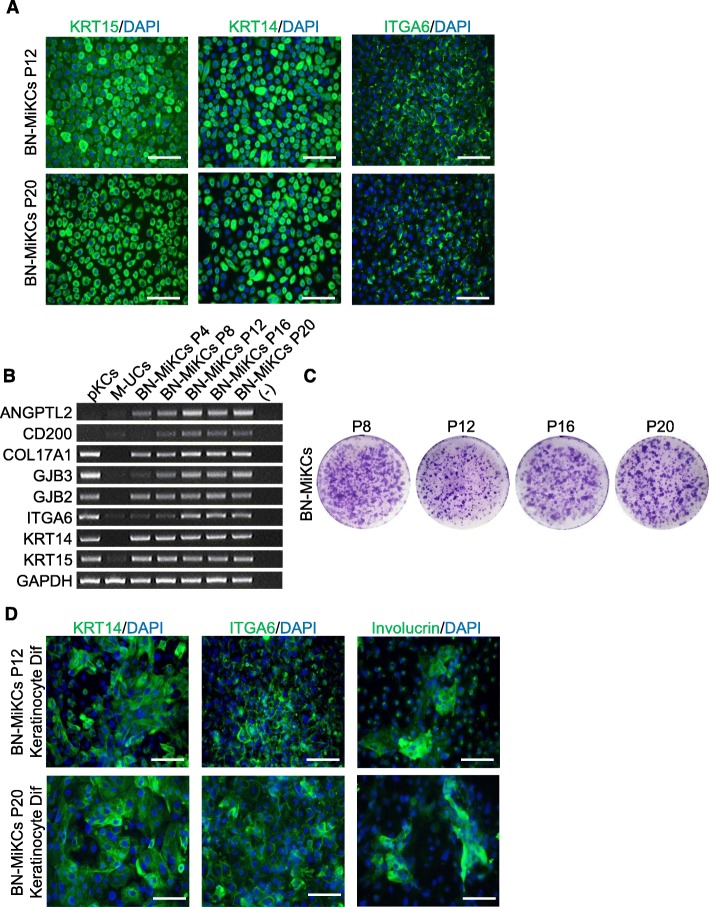
Long-term propagation and characterization of BN-MiKCs. **a** Immunofluorescence analysis of stem cell markers (KRT15, KRT14, and ITGA6) in BN-MiKCs at passage 12 and 20. Scale bars = 200 μm. **b** mRNA expression of keratinocyte stem cell-specific (Col17A1, GJB3, GJB2, ITGA6, KRT14, and KRT15) and HFSC-specific (ANGPTL2 and CD200) markers in pKCs, M-UCs, and BN-MiKCs at passage 4, 8, 12, 16, and 20. **c** Colony-forming assay of BN-MiKCs at passage 8, 12, 16, and 20. BN-iKCs were seeded at a density of 0.25 × 10^4^ cells per well in a 6-well plate with a 3 T3-J2 feeder layer and cultured for 1 week. The plates were stained with crystal violet. **d** Expression of keratinocyte markers (ITGA6, KRT14, and Involucrin) in mature keratinocytes differentiated from BN-MiKCs at passage 12 and 20. Scale bars = 200 μm. Nuclei were counterstained with DAPI

To assess whether BN-MiKCs and BN-FiKCs retained a long-term self-renewal potential, we cultured them in KSFM for at least 20 passages. These cells exhibited robust expression of typical stem cell markers (Fig. 5a, b, and S8D–F). In contrast with culture in the presence of FBS, KSFM contributed to stable colony formation and preservation of the differentiation potential of BN-MiKCs and BN-FiKCs during long-term expansion (Fig. 5c, d, S8G, and S9A, B). Overall, these results demonstrate that iKCs can be generated and expanded in serum- and feeder cell-free culture conditions.

### Global gene expression profiles of BN-iKCs

**Fig. 6:**
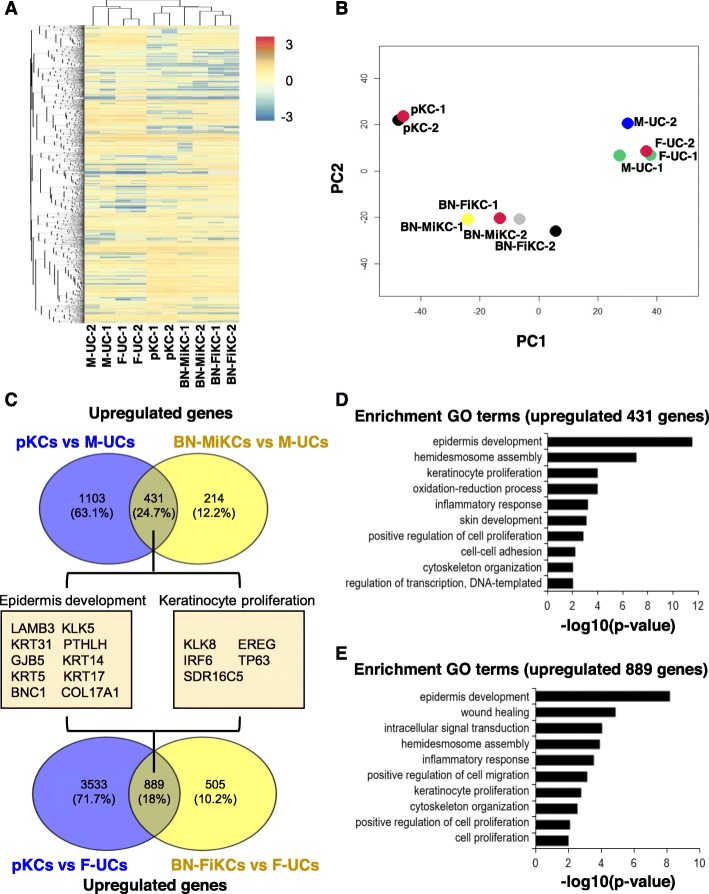
Global gene expression profiling of BN-iKCs. **a** Heat map with hierarchical clustering of genes (FDR < 0.05) in M-UCs, F-UCs, pKCs, BN-MiKCs and BN-FiKCs based on RNA-sequencing analysis. Red and blue in the heat map indicate upregulated and downregulated genes, respectively. **b** principal component analysis (PCA) of RNA-seq data from M-UCs, F-UCs, pKCs, BN-MiKCs and BN-FiKCs. **c** Venn diagram showing the numbers of significantly upregulated genes (FDR < 0.05) in the pKCs vs. M-UCs and BN-MiKCs vs. M-UCs comparisons (upper) and pKCs vs. F-UCs and BN-FiKCs vs. F-UCs comparisons (lower). **d** and **e** GO analysis of overlapping upregulated genes from (**c**)

We compared the global transcriptome profile of human urine cell-derived iKCs with those of parental urine cells and pKCs by RNA-sequencing. Hierarchical clustering (FDR < 0.05) showed the genome-wide conversion of M- and F-UCs into BN-MiKCs and BN-FiKCs, respectively, and these cells exhibited a high degree of similarity to pKCs (Fig. 6a). Moreover, the principal component analysis (PCA) indicated that BN-iKCs successfully separated from UCs and more closed to pKCs (Fig. 6b). Next, we analyzed significantly changed genes (FDR < 0.05) in four comparisons (BN-MiKCs vs. M-UCs, pKCs vs. M-UCs, BN-FiKCs vs. F-UCs, and pKCs vs. F-UCs) by using Venn diagram: 645, 1534, 1449, and 4422 genes were upregulated, while 509, 1965, 1098 and 4008 genes were downregulated, respectively (Fig. 6c and S10A). Compared with parental urine cells, 431 and 429 genes were upregulated and downregulated, respectively, in both BN-MiKCs and pKCs, and 889 and 919 genes were upregulated and downregulated, respectively, in both BN-FiKCs and pKCs. Gene ontology (GO) categories related to epidermis processes, including epidermis development, hemidesmosome assembly and keratinocyte proliferation, were significantly enriched in BN-iKCs and pKCs, while genes related on extracellular matrix organization, ureteric bud development and angiogenesis were highly downregulated (Fig. 6d, e and S10 B, C, D, E).

### Reconstitution of a human skin equivalent in vitro

**Fig. 7:**
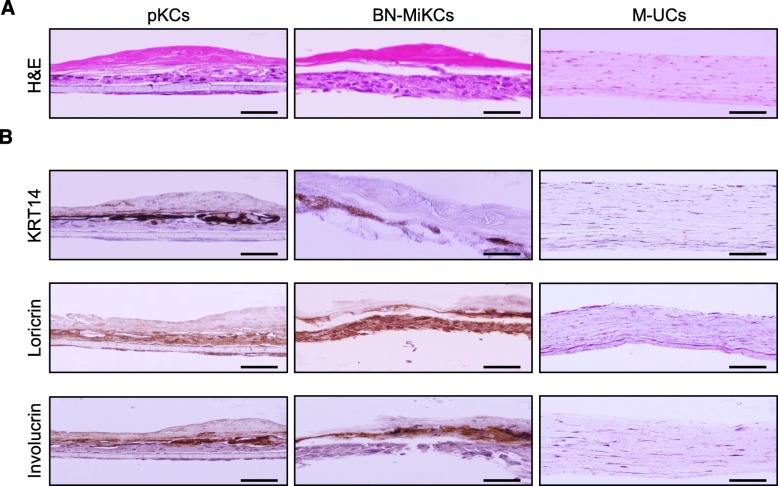
In vitro reconstruction of a 3D skin equivalent. **a** H&E staining of skin equivalents reconstituted by pKCs, BN-MiKCs, and M-UCs. Scale bars = 100 μm. **b** Expression of an epidermis basal layer marker (KRT14) and differentiated keratinocyte markers (Loricrin and Involucrin) in skin equivalents generated by pKCs, BN-MiKCs, and M-UCs. Scale bars = 100 μm

Upon culture on a layer of dermal fibroblasts with collagen under air-liquid interface conditions, epidermal keratinocytes form stratified sheets and differentiate to establish a three-dimensional (3D) skin equivalent [27, 28]. Skin equivalents reconstituted from patient- and disease-specific cells are a powerful tool to elucidate the molecular processes that affect skin physiology and pathophysiology, to investigate various skin diseases, drug discovery, and therapeutic applications, and to perform skin grafting [48, 49]. Accordingly, we investigated whether BN-iKCs can reconstitute skin equivalents comparably to pKCs. The skin equivalents generated by BN-iKCs were morphologically similar to those generated by pKCs, and they expressed the basal and suprabasal layer marker KRT14 as well as epidermal differentiation markers such as Involucrin and Loricrin (Fig. 7). As expected, urine cells did not form epidermis-like structures and did not express epidermal markers (Fig. 7). These results demonstrate the tissue-regenerative capacity of BN-iKCs and their progeny, implying that our approach for generating and expanding iKCs could be translated from the bench to the bedside.

### Overexpression of BN allowed fibroblasts to express keratinocyte stem genes

To evaluate whether other somatic cell sources are exploited to generate iKCs by overexpression of BN. Human foreskin fibroblasts were transduced with retroviruses encoding BN. These BN-overexpressed fibroblasts exhibited expression of the keratinocyte stem markers in mRNA and protein levels (Figure S11). However, these cells showed a very low reprogramming efficiency and failed in the formation of colonies, suggesting that the choice of the somatic cell source is one of the key drivers for successful direct lineage conversion.

## Discussion

Here, we describe a novel method to directly reprogram human urine cells into long-term expandable iKCs, allowing in vitro reconstitution of personalized skin. This is achieved using two lineage-specific transcription factors, namely, BN, and culture in feeder cell- and serum-free conditions. BN-driven iKCs were similar to pKCs in terms of their morphology, protein expression, 2D and 3D differentiation potential, and global gene expression profile. Our approach has several advantages over existing methods for clinical trials. First, the present protocol reprograms human urine cells, allowing patient-specific therapies to be conducted in a non-invasive manner. Second, direct reprogramming of functional cell types from one lineage to another is a critical tool for clinical trials because it bypasses an intermediate pluripotent stage and thereby minimizes the risk of tumorigenesis after transplantation [50]. Third, compared with iPSC-based approaches using the canonical reprogramming factors OCT4, SOX2, KLF4, and cMYC [51–54], the poor proliferative ability of functional cells converted using lineage-specific transcription factors is a major challenge. The reprogramming strategy into proliferative stem/progenitor cells and acquisition of expandable transient intermediates can generate a larger amount of material for biomedical applications, while maintaining all the therapeutic benefits [4]. In addition, stem/progenitor cells are more desirable for transplantation due to their efficient engraftment and better integration in vivo [55]. Fourth, the generated iKCs are extensively expandable in serum- and feeder cell-free systems. Previous methods for in vitro expansion of epithelial stem cells rely on co-culture with mouse fibroblasts in serum-containing basal medium, which contains undefined and variable mixtures of molecules [39]. In the last decades, substantial efforts have been made to isolate and culture epithelial stem cells in serum-free medium and to retain their undifferentiated state in feeder cell-free conditions [56–58]. Nonetheless, it remains challenging to develop culture conditions that address the safety concerns of regulatory affairs. Finally, the in vitro reconstitution of human skin demonstrated the similarities between the generated iKCs and their in vivo counterparts (pKCs) as well as their regenerative ability, which is a hallmark of stem cells. Although this has been achieved by isolation of epidermal stem cells, a skin equivalent derived from human iKCs has not been previously reported.

Despite the improved expansion of iKCs and successful construction of a human skin equivalent in vitro, clinicians have continually questioned their value versus the risks of retrovirus-mediated gene transfer, which can potential contribute to undesired genotoxicity, insertional mutagenesis, and tumorigenesis [59]. Various integration-free approaches have been developed to convert cells into iPSCs and adult stem/progenitor cells, including viral (adenoviruses, Sendai viruses, and Cre-excisable viruses) [60–63] and non-viral (DNA expression vectors, minicircle vectors, and episomal vectors) [64–67] vectors and non-DNA-based systems (proteins, mRNAs, and chemicals) [68–73]. Thus, further studies of genetic delivery that avoids potential issues associated with viral integration are required to increase the feasibility of clinical trials. Meanwhile, we found that exogenous △NP63α was continuously expressed in BN-iKCs, while its endogenous expression were not detected, which is consistent with a previous study [12]. △NP63α plays critical roles in maintaining the self-renewal and proliferation capacities of keratinocyte stem/progenitor cells and regulating their differentiation for stratification of the epidermis [74, 75]. Antonini et al. demonstrated that △Np63γ robustly induces endogenous expression of △NP63α in HeLa cells [76]. Another study reported that P53 is a potent transcriptional activator of △NP63α in immortalized mammary epithelial cells [77]. Collectively, gene-free methodology for activating endogenously expressed △NP63α, mediated by genes such as △Np63γ or P53, may be a promising alternative to virus-mediated gene transfer.

In the present study, BN-iKCs expressed HFSC-specific markers (CD200 and ANGPTL2) and successfully differentiated into sebocytes. Nonetheless, hair follicles were not found in nude mice subcutaneously injected with BN-iKCs and mouse neonatal dermis cells (data not shown). Based on previous studies [78–80], this inability to induce hair follicle formation may be explained by repression of HFSC markers such as LHX2, LGR5, and LGR6, which contribute to preservation of the self-renewal ability of stem cells and fate specification to whole hair follicles. Thus, BN might only drive reprogramming of human urine cells into iKCs with a partial HFSC phenotype. Generation of iKCs with a full HFSC phenotype may require genes that play essential roles in regulation of hair follicle morphogenesis and specification (i.e., SOX9, TCF3, TCF4, and SNAI2) [81–83]. Moreover, accumulating evidence indicates that epigenetic factors, including ACTL6a, DNMT1, HDAC1, HDAC2, and SUZ12, help to maintain stemness and/or suppress differentiation of HFSCs [84–87]. Additional transcription and epigenetic factors could therefore be useful for the acquisition of HFSC properties during induction of iKCs.

## Conclusions

We demonstrated that human urine cells can be directly reprogrammed into expandable iKCs using a defined set of lineage-specific transcription factors, namely, BN. In addition, the generated iKCs could undergo long-term expansion in serum- and feeder cell-free conditions, and, more importantly, self-assembled to regenerate a fully stratified epithelium sheet in vitro. Further studies for generating integration-free and hair folliculogenic iKCs could offer enormous promise for clinical applications. Nevertheless, this study describes a novel method to generate human iKCs that can undergo long-term expansion, which will facilitate their broad applicability in an efficient and patient-specific manner. In addition, these cells could be used as an in vitro platform to explore the cellular and molecular cues governing skin regeneration and to address scientific or medical questions in dermatology and skin biology.

## Supplementary information

**Additional file 1 Table S1.** Antibody information used in Immunofluorescence and Immunohistochemistry. **Table S2**. Primer information used in RT-PCR and Real-Time PCR. **Figure S1.** Immunofluorescence analysis of keratinocyte lineage markers. **Figure S2.** Induction of KCs from human urine cells using several combinations of transcription factors. **Figure S3.** Selection and further expansion of induced urine cells using several combinations of transcription factors. **Figure S4.** Differentiation of human urine cells into terminally differentiated KCs. **Figure S5.** Differentiation into sebocytes. **Figure S6.** Direct reprogramming of F-UCs into BN-iKCs. **Figure S7.** Selection of BN-iKC colonies derived from BN-overexpressed UCs. **Figure S8.** Generation and long-term culture of BN-iKCs derived from F-UCs in serum-free conditions. **Figure S9.** Differentiation potential of BN-FiKCs. **Figure S10.** RNA-sequencing analysis of BN-iKCs. **Figure S11.** Reprogramming of fibroblasts into iKCs by using BN.

## Data Availability

All data generated in the current study are available from the corresponding author on reasonable request. RNA-sequencing data has been submitted and deposited in Gene Expression Omnibus (GEO) under accession number GSE129316.

## Abbreviations

iKC: induced keratinocyte like cell
B: BMI1
N: △NP63α
K: KLF4
iPSC: induced pluripotent stem cell
EMT: Epithelial-mesenchymal transition
CFE: Colony-forming efficiency
HFSC: Hair follicle epithelial cell
KSFM: Keratinocyte serum free medium
