# Supplementary material for: Long-term expansion of directly reprogrammed keratinocyte-like cells and in vitro reconstitution of human skin
Source: J Biomed Sci. 2020 Apr 20;27:56. doi: 10.1186/s12929-020-00642-1 (PMC7171822; doi:10.1186/s12929-020-00642-1)
Supplement: Supplementary file 1 — Additional file 1 Table S1. Antibody information used in Immunofluorescence and Immunohistochemistry. Table S2. Primer information used in RT-PCR and Real-Time PCR. Figure S1. Immunofluorescence analysis of keratinocyte lineage markers. Figure S2. Induction of KCs from human urine cells using several combinations of transcription factors. Figure S3. Selection and further expansion of induced urine cells using several combinations of transcription factors. Figure S4. Differentiation of human urine cells into terminally differentiated KCs. Figure S5. Differentiation into sebocytes. Figure S6. Direct reprogramming of F-UCs into BN-iKCs. Figure S7. Selection of BN-iKC colonies derived from BN-overexpressed UCs. Figure S8. Generation and long-term culture of BN-iKCs derived from F-UCs in serum-free conditions. Figure S9. Differentiation potential of BN-FiKCs. Figure S10. RNA-sequencing analysis of BN-iKCs. Figure S11. Reprogramming of fibroblasts into iKCs by using BN. [file 12929_2020_642_MOESM1_ESM.pdf]

Table S1. Antibody information used in Immunofluorescence and Immunohistochemistry

Antibody	Company	Host	Cat. No.
KRT15	Invitrogen	Mouse	MA1-90929
ITGA6	Invitrogen	Rat	14-0495-82
KRT14	Abcam	Rabbit	ab15461
KRT10	Santa Cruz	Mouse	sc-23877
Involucrin	Invitrogen	Mouse	MA5-11803
Loricrin	Abcam	Rabbit	ab85679

Table S2. Primer information used in RT-PCR and Real-Time PCR

Gene	Primer	Sequence
ANGPTL2	Forward	TACATGGCACAACGGCAAGCA
	Reverse	TTGGAGTGGGCACAGGCGTTAT
CD200	Forward	AATGGGACCACGTCTGTTAC
	Reverse	GCGGAACTGAAAACCAATAGC
ITGA6	Forward	TGCACGCGGATCGAGTTTGA
	Reverse	AACACCGCCCAAAGATGTCTCG
KRT15	Forward	TGACATAAAGACACGGCTGG
	Reverse	TTGATGTGGAAATTGCTGCTG
KRT14	Forward	TGTGGAAGCCGACATCAATG
	Reverse	CTCTCAGGGCATTCATCTCC
GJB2	Forward	AGGAGATCAAAACCCAGAAGG
	Reverse	AAGACGTACATGAAGGCGG
GJB3	Forward	TGTACGACAACGCAGGCAAGA
	Reverse	ACCAGGCGCGGCATATTGAA
KRT8	Forward	CTCAGAGATCAACCGGAACA
	Reverse	TTCATCAGCTCCTGGTACTCAC
KRT18	Forward	AGACCATGCAAAGCCTGAAC
	Reverse	GCAGTCGTGTGATATTGGTGTC
KRT1	Forward	TGGCTCTGCTGGGATCATCAACT
	Reverse	CCGACTTCCAAATCCACCACCA
Invocurin	Forward	TCCCAGTGGAGGTCCCATCAAA
	Reverse	ATGCTGTTCCCAGTGCTGTTGCT
Filaggrin	Forward	TTCGGCAAATCCTGAAGAATC
	Reverse	CTTGAGCCAACTTGAATACCATC
COL17A1	Forward	CCATCACAGGCGAGACTTTC
	Reverse	GCCCATCAAGTACTGACGTAG
GAPDH	Forward	GTGGTCTCCTCTGACTTCAACA
	Reverse	CTCTTCCTCTTGTGCTCTTGCT

Supplementary Figure Captions

Figure S1. Immunofluorescence analysis of keratinocyte lineage markers. (A) Phase-contrast image and immunostaining analysis of stem cell markers (KRT15 and ITGA6), a basal/suprabasal marker (KRT14), and differentiated markers (KRT10 and Involucrin) in urine cells. Scale bars=500 µm. **(B)** Phase-contrast image and immunostaining analysis of stem cell markers (KRT15 and ITGA6) in 3T3-J2 feeder cells. Scale bars=500 µm. **(C)** Phase-contrast image and immunostaining analysis of stem cell markers (KRT15 and ITGA6), a basal/suprabasal marker (KRT14) and differentiated markers (KRT10 and Involucrin) in pKCs. Nuclei were counterstained with DAPI. Scale bars=200 µm.

Figure S2. Induction of KCs from human urine cells using several combinations of transcription factors. (A) At day 12 post-induction, urine cells (left) acquired a keratinocyte-like morphology upon overexpression of NK (right). Scale bars=200 µm. **(B)** Expression of exogenous mRNAs in BNK-, BN-, BK-, NK-, B-, N- and K-infected cells, pKCs and urine cells at day 12 post-induction. **(C)** Immunostaining of B-, N- and K-infected cells and urine cells with specific antibodies against the stem cell markers KRT15 and ITGA6 at day 12 post-induction. Nuclei were counterstained with DAPI. The lower row shows magnified images of the boxed areas in the upper row. Scale bars=200 µm (upper), 100 µm (lower).

Figure S3. Selection and further expansion of induced urine cells using several combinations of transcription factors. (A–C) Selection of ITGA6^{+}/KRT15^{+} cells derived from **(A)** BNK21-11, **(B)** NK16-6 and **(C)** N20-10 cells by single-colony picking. Selected cells at passage 3 were stained with specific antibodies against ITGA6 and KRT15. Nuclei were counterstained with DAPI. Images on the right are magnified views of the boxed

areas in the central images. Scale bars=200 μm. **(D)** Expression of exogenous mRNAs in urine cells, selected BN28-2, BN28-5 and BN28-6. **(E)** Phase-contrast images of BN28-6, BNK21-11, NK16-6 and N20-10 cells at passage 4. Scale bars=200 μm. **(F)** Colony-forming assay of BNK21-11 cells during long-term expansion. BNK21-11 cells were seeded at a density of 0.25×10^4 cells per well in a 6-well plate with a 3T3-J2 feeder layer and cultured for 1 week. The plates were stained with crystal violet.

Figure S4. Differentiation of human urine cells into terminally differentiated KCs. (A–C) Phase-contrast image and expression of **(A)** a stem cell marker (KRT15) and **(B and C)** mature keratinocyte markers (KRT10 and Involucrin) in mature keratinocyes differentiated from urine cells. Nuclei were counterstained with DAPI. Scale bars=200 μm.

Figure S5. Differentiation into sebocytes. (A–F) Oil red O staining of **(A)** pKCs, **(B)** BN28-6 cells, **(C)** sebocytes differentiated from BN28-5 cells, **(D)** sebocytes differentiated from BN28-2 cells, **(E)** urine cells and **(F)** sebocytes differentiated from urine cells. Images on the right are magnified views of the boxed areas in images on the left. Scale bars=200 μm.

Figure S6. Direct reprogramming of F-UCs into BN-iKCs. (A) Phase-contrast images of F-UCs and BN-infected F-UCs at day 12 post-induction. Scale bars=500 μm. **(B)** RT-PCR analysis of stem cell markers (Col17A1, GJB3, GJB2, ITGA6, KRT14 and KRT15) in pKCs, F-UCs and ITGA6^{+}KRT15^{+} BN-iKCs (BN328-1) selected by single-colony picking. **(C)** Immunostaining analysis of stem cell markers (KRT15, KRT14 and ITGA6) in BN328-1 cells. Nuclei were counterstained with DAPI. Scale bars=200 μm. **(D)** Immunostaining analysis of keratinocyte markers (ITGA6, KRT14 and KRT10) in mature keratinocytes differentiated from BN328-1 cells.

Nuclei were counterstained with DAPI. Scale bars=200 μm.

Figure S7. Selection of BN-iKC colonies derived from BN-overexpressed UCs.

(A) Expression of exogenous △NP63α and Bmi1 in M-UCs and selected BN-MiKCs. **(B)** Expression of exogenous △NP63α and Bmi1 in M-UCs and selected BN-FiKCs. **(C)** At the same passage number 12, comparison of exogenous △NP63α expression of M-UCs, BN-MiKCs, mature keratinocytes differentiated from BN-MiKCs, F-UCs, BN-FiKCs and mature keratinocytes differentiated from BN-FiKCs. **(D)** Immunofluorescence analysis of basal/suprabasal marker (KRT14), terminal differentiated keratinocyte markers (Involucrin and Loricrin) and early differentiated keratinocyte marker (KRT10) in PMA-treated BN-MiKCs and BN-FiKCs at passage 12. Nuclei were counterstained with DAPI. Scale bars=200 μm.

Figure S8. Generation and long-term culture of BN-iKCs derived from F-UCs in serum-free conditions. (A) Phase-contrast images of $ITGA6^{+}KRT15^{+}$ BN-FiKCs selected by single-colony picking. Scale bars=200 μm. **(B)** RT-PCR analysis of stem cell markers (Col17A1, GJB3, GJB2, ITGA6, KRT14 and KRT15) in pKCs, F-UCs and BN-FiKCs. **(C–E)** Expression of stem cell markers (KRT15, KRT14 and ITGA6) in **(C)** BN-FiKCs at passage 4, **(D)** BN-FiKCs at passage 12 and **(E)** BN-FiKCs at passage 20. Nuclei were counterstained with DAPI. Scale bars=200 μm. **(F)** RT-PCR analysis of stem cell markers (GJB2, KRT14 and KRT15) in pKCs, F-UCs and BN-FiKCs at passage 2, 6 and 20. **(G)** Colony-forming assay of BN-FiKCs during long-term expansion. BN-FiKCs were seeded at a density of 0.25×10^{4} cells per well in a 6-well plate with a 3T3-J2 feeder layer and cultured for 1 week. The plates were stained with crystal violet.

Figure S9. Differentiation potential of BN-FiKCs. (A) Immunofluorescence analysis of keratinocyte markers

(ITGA6, KRT14, KRT10 and Involucrin) in mature keratinocytes differentiated from BN-FiKCs at passage 12. Scale bars=200 μm. **(B)** Immunofluorescence analysis of keratinocyte markers (ITGA6, KRT14 and Involucrin) in mature keratinocytes differentiated from BN-FiKCs at passage 20. v **(C)** Oil red O staining of sebocytes differentiated from BN-FiKCs. The image on the right is a magnified view of the boxed area in the image on the left. Scale bars=200 μm.

Figure S10. RNA-sequencing analysis of BN-iKCs. (A) Venn diagram showing the numbers of significantly downregulated genes (FDR<0.05) in the pKCs vs. M-UCs and BN-MiKCs vs. M-UCs comparisons (upper) and pKCs vs. F-UCs and BN-FiKCs vs. F-UCs comparisons (lower). **(B and C)** GO analysis of overlapping downregulated genes from **(A). (D and E)** Heat map with hierarchical clustering of genes (Top100) whose expression was significantly changed (FDR<0.05) between M-UCs and BN-MiKCs (D), F-UCs and BN-FiKCs (E).

Figure S11. Reprogramming of fibroblasts into iKCs by using BN. (A) RT-PCR analysis of stem cell markers (KRT14, KRT15 and ITGA6) in pKCs, FBs and BN-FBs. **(B)** Expression of stem cell markers (KRT15 and KRT14) in FBs and BN-FBs. FB, Fibroblast; Nuclei were counterstained with DAPI. Scale bars=200 μm.

Figure S1

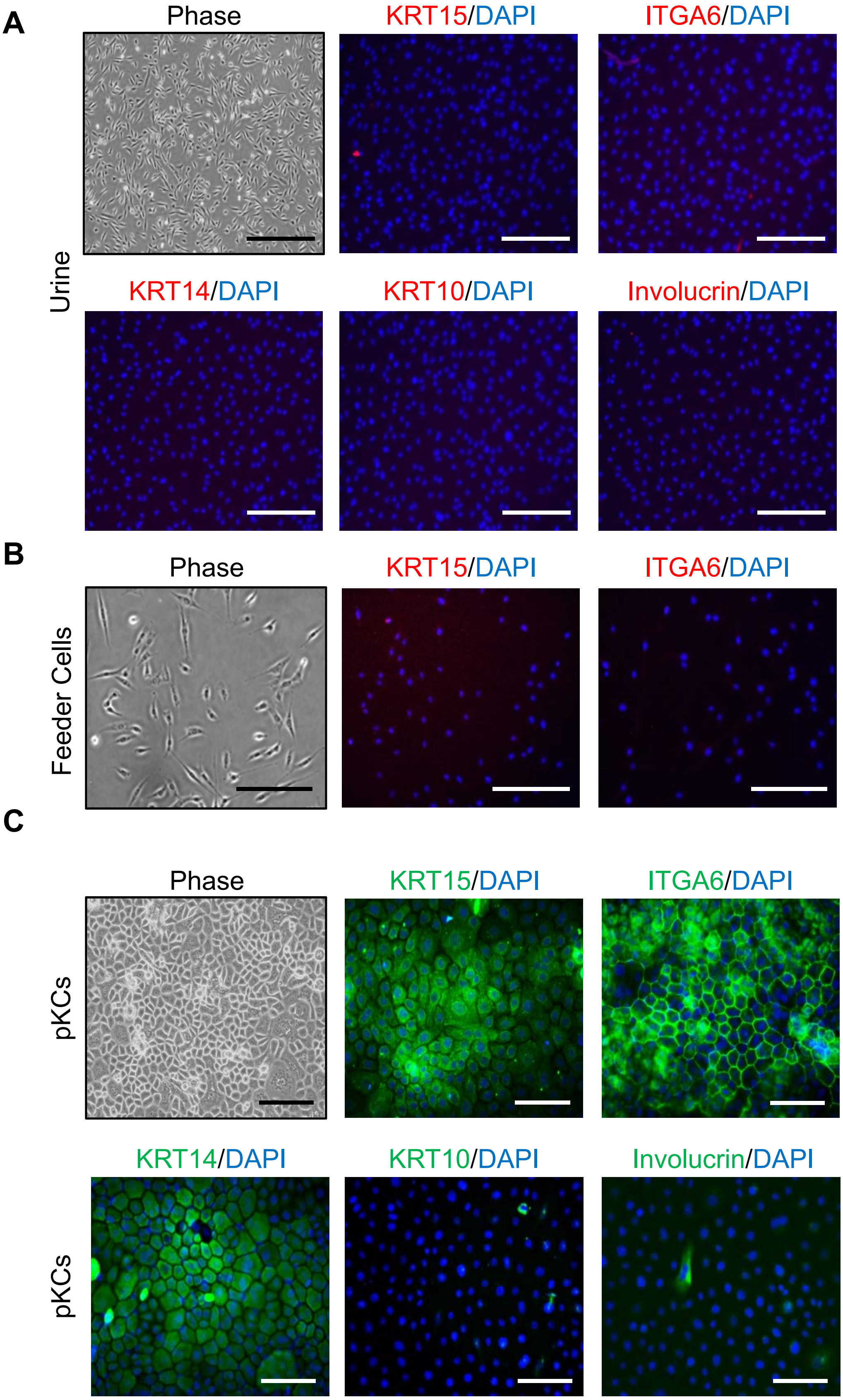

A
Phase
KRT15/DAPI
ITGA6/DAPI
Urine
KRT14/DAPI
KRT10/DAPI
Involucrin/DAPI
B
Phase
KRT15/DAPI
ITGA6/DAPI
Feeder Cells
C
Phase
KRT15/DAPI
ITGA6/DAPI
pKCs
KRT14/DAPI
KRT10/DAPI
Involucrin/DAPI
pKCs

Figure S2

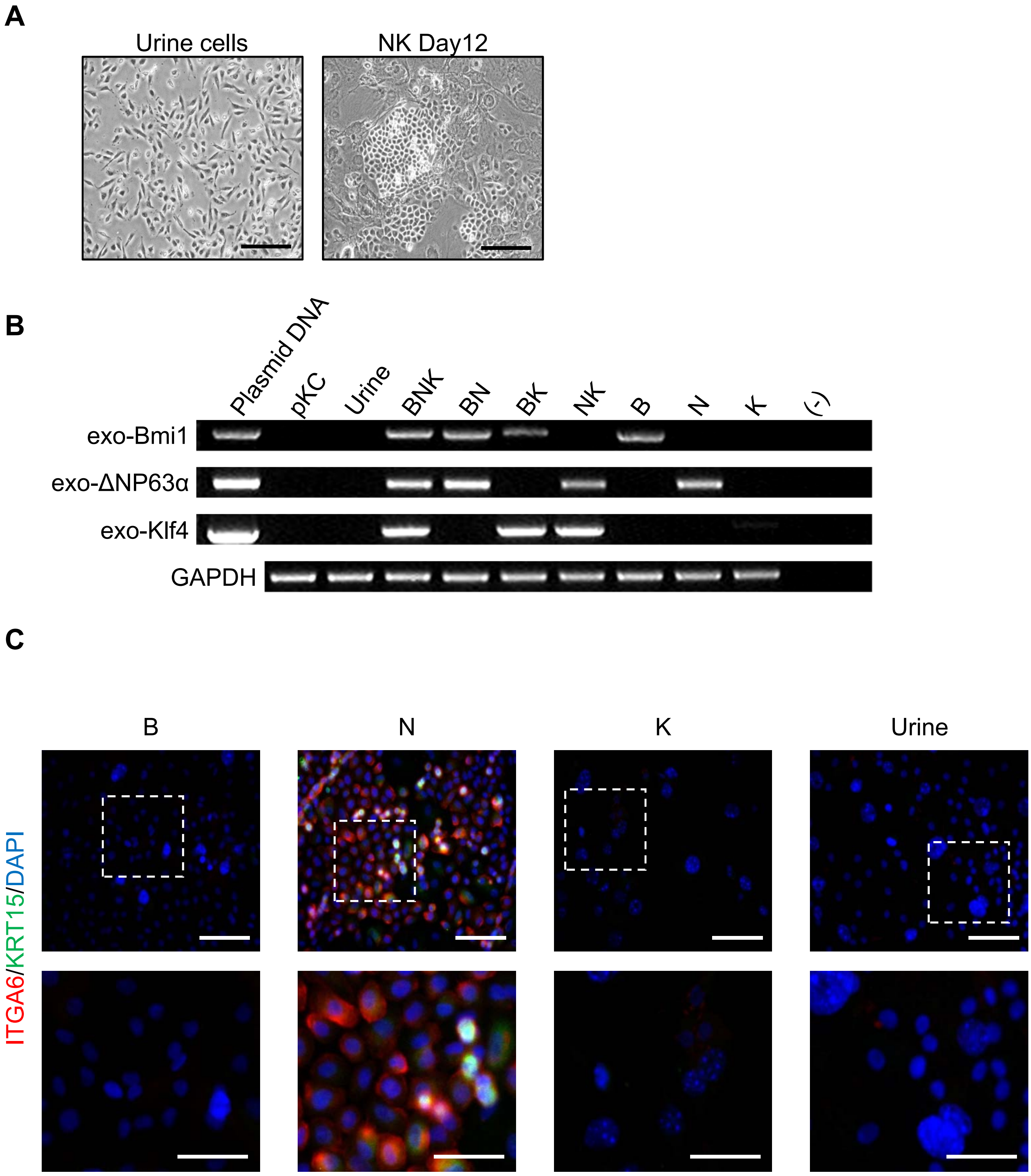
A
Urine cells
NK Day12
B
Plasmid DNA
pKC
Urine
BNK
BN
BK
NK
B
N
K
(-)
exo-Bmi1
exo-ΔNP63α
exo-Klf4
GAPDH
C
B
N
K
Urine
ITGA6/KRT15/DAPI

Figure S3

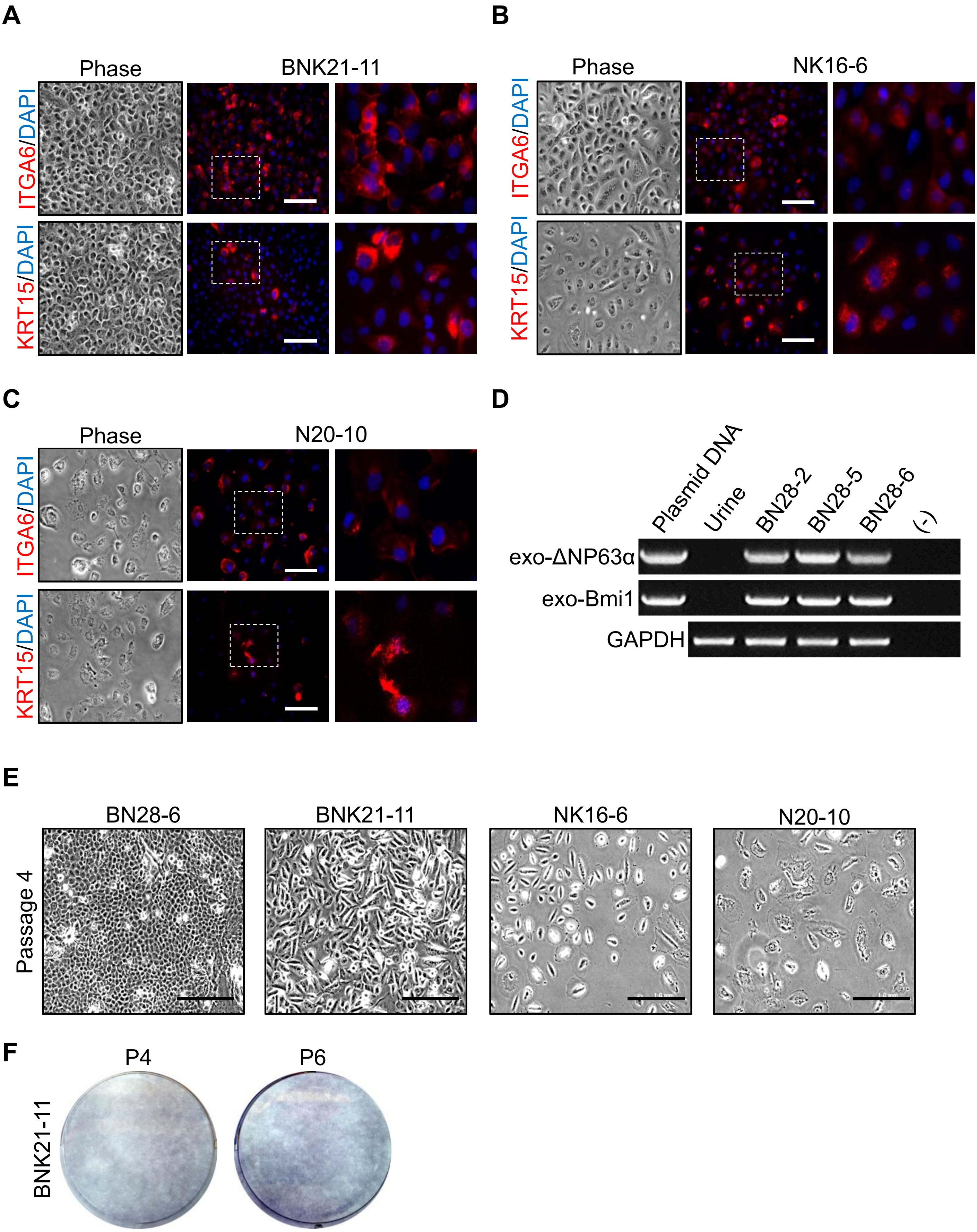
A
Phase
BNK21-11
ITGA6/DAPI
KRT15/DAPI
B
Phase
NK16-6
ITGA6/DAPI
KRT15/DAPI
C
Phase
N20-10
ITGA6/DAPI
KRT15/DAPI
D
Plasmid DNA
Urine
BN28-2
BN28-5
BN28-6
(-)
exo-ΔNP63α
exo-Bmi1
GAPDH
E
BN28-6
BNK21-11
NK16-6
N20-10
Passage 4
F
P4
P6
BNK21-11

Figure S4

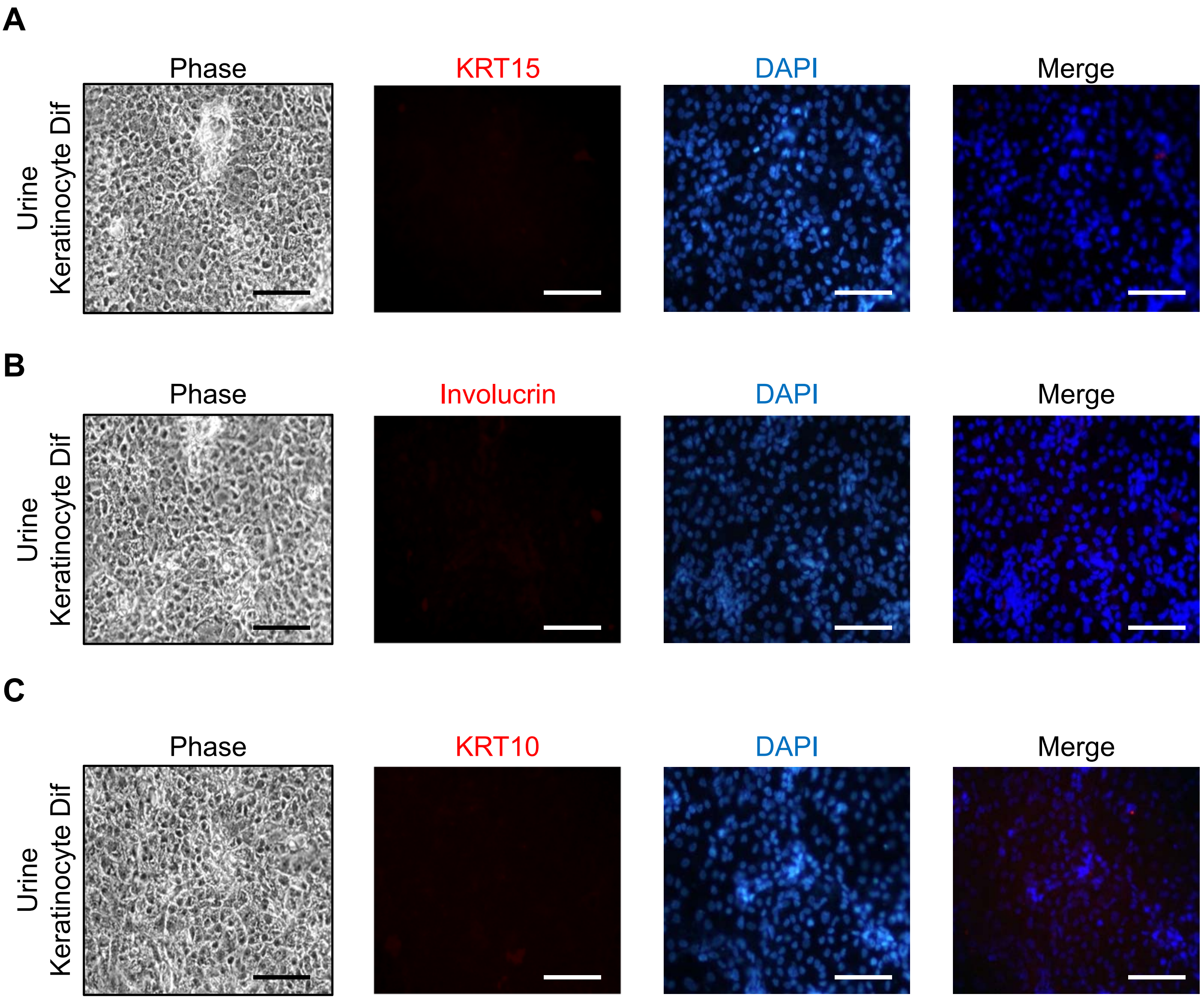
A
Phase
KRT15
DAPI
Merge
Urine Keratinocyte Dif
B
Phase
Involucrin
DAPI
Merge
Urine Keratinocyte Dif
C
Phase
KRT10
DAPI
Merge
Urine Keratinocyte Dif

Figure S5

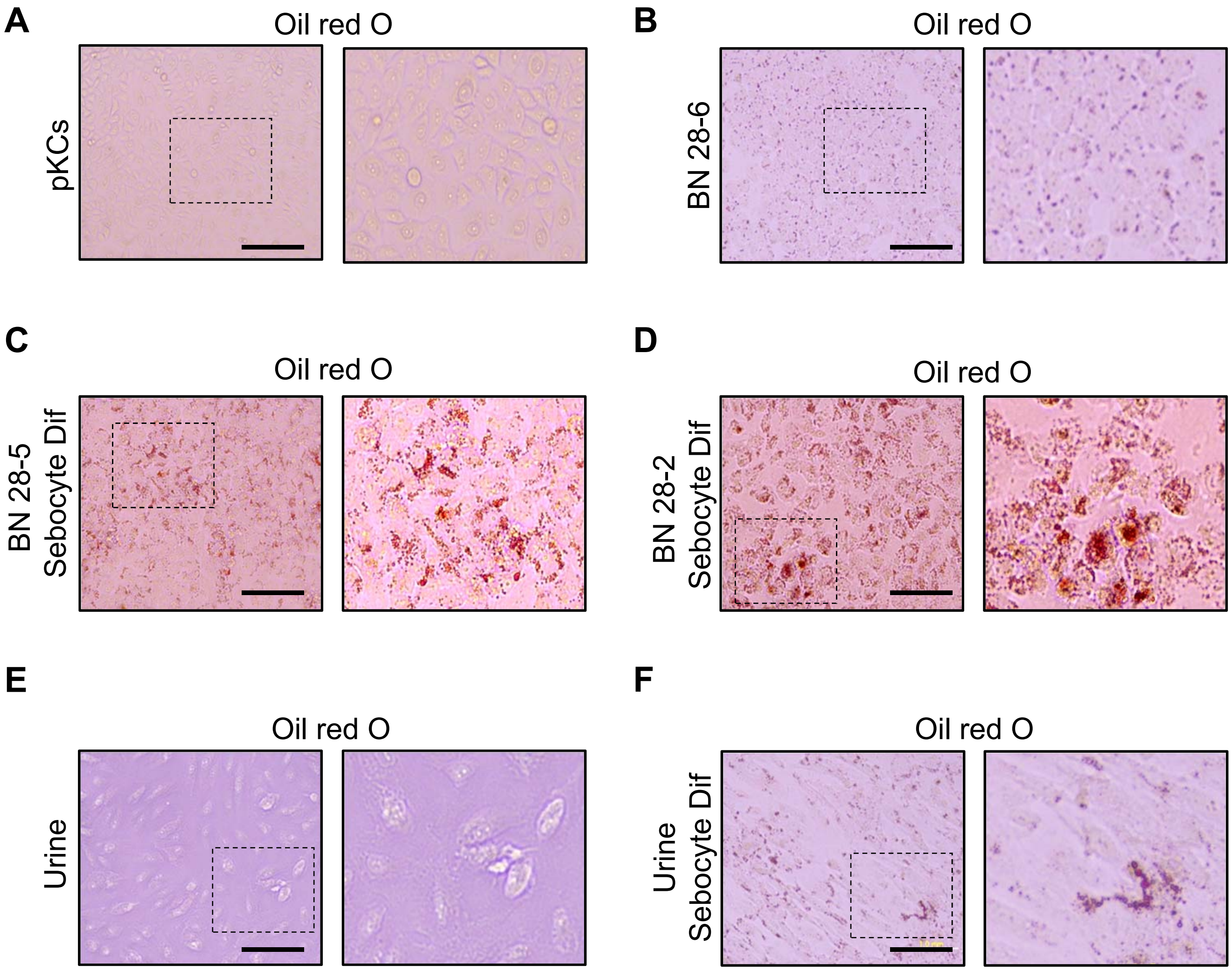
A
Oil red O
pKCs
B
Oil red O
BN 28-6
C
Oil red O
BN 28-5
Sebocyte Dif
D
Oil red O
BN 28-2
Sebocyte Dif
E
Oil red O
Urine
F
Oil red O
Urine
Sebocyte Dif

Figure S6

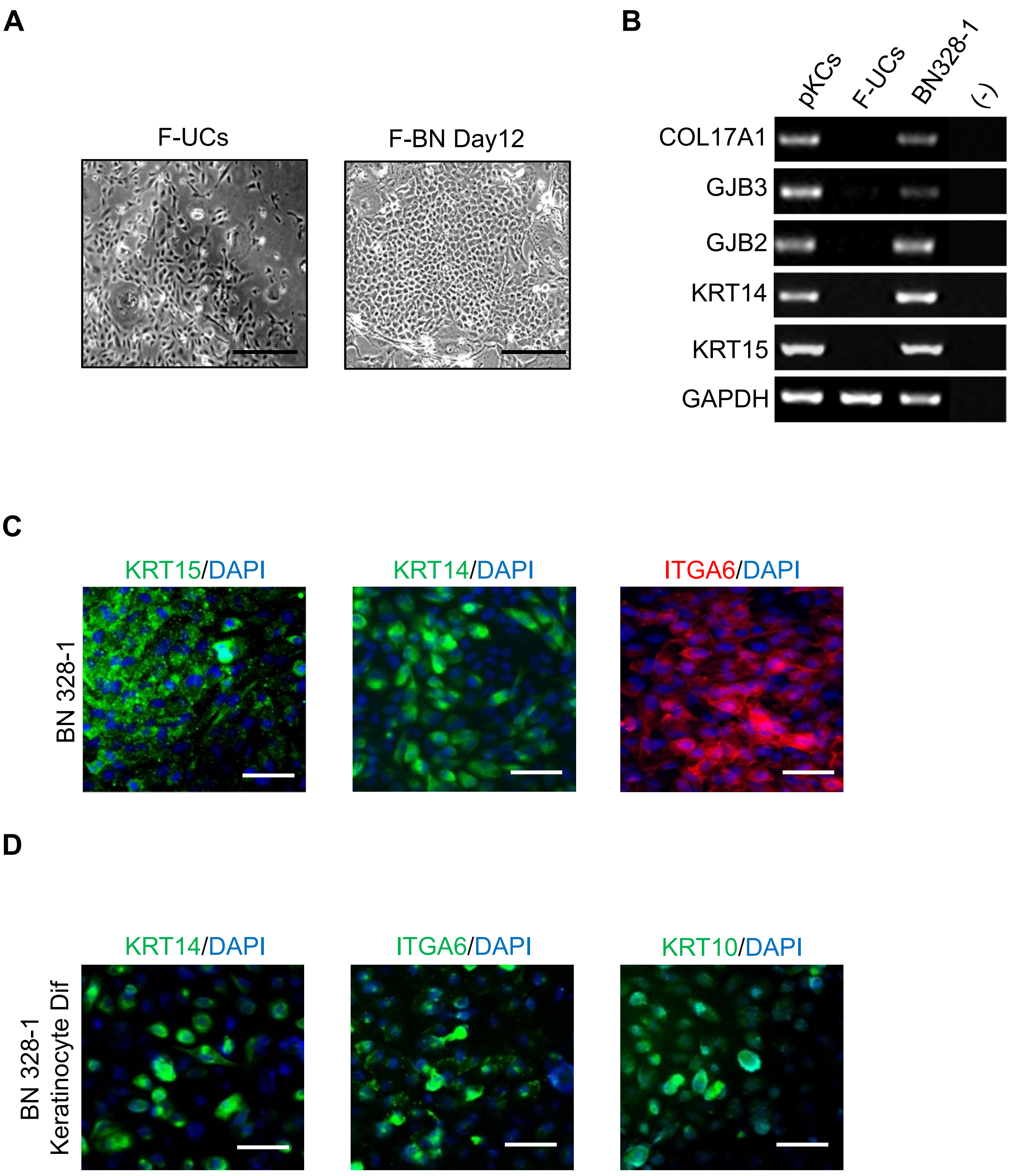
A
F-UCs
F-BN Day12
B
pKCs
F-UCs
BN328-1
(-)
COL17A1
GJB3
GJB2
KRT14
KRT15
GAPDH
C
KRT15/DAPI
KRT14/DAPI
ITGA6/DAPI
BN 328-1
D
KRT14/DAPI
ITGA6/DAPI
KRT10/DAPI
BN 328-1
Keratinocyte Dif

Figure S7

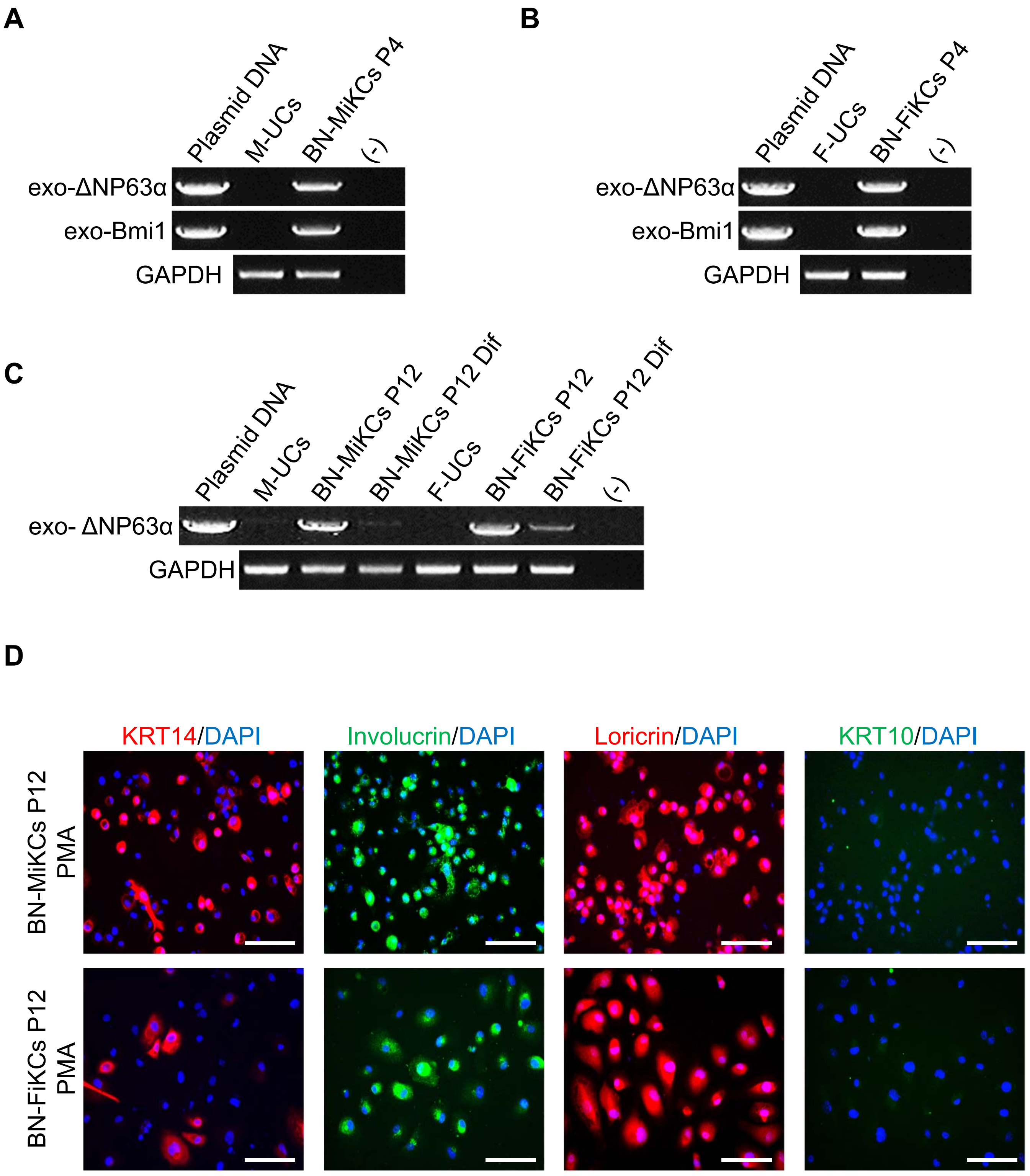

A
Plasmid DNA
M-UCs
BN-MiKCs P4
(-)
exo-ΔNP63α
exo-Bmi1
GAPDH
B
Plasmid DNA
F-UCs
BN-FiKCs P4
(-)
exo-ΔNP63α
exo-Bmi1
GAPDH
C
Plasmid DNA
M-UCs
BN-MiKCs P12
BN-MiKCs P12 Dif
F-UCs
BN-FiKCs P12
BN-FiKCs P12 Dif
(-)
exo- ΔNP63α
GAPDH
D
KRT14/DAPI
Involucrin/DAPI
Loricrin/DAPI
KRT10/DAPI
BN-MiKCs P12
PMA
BN-FiKCs P12
PMA

Figure S8

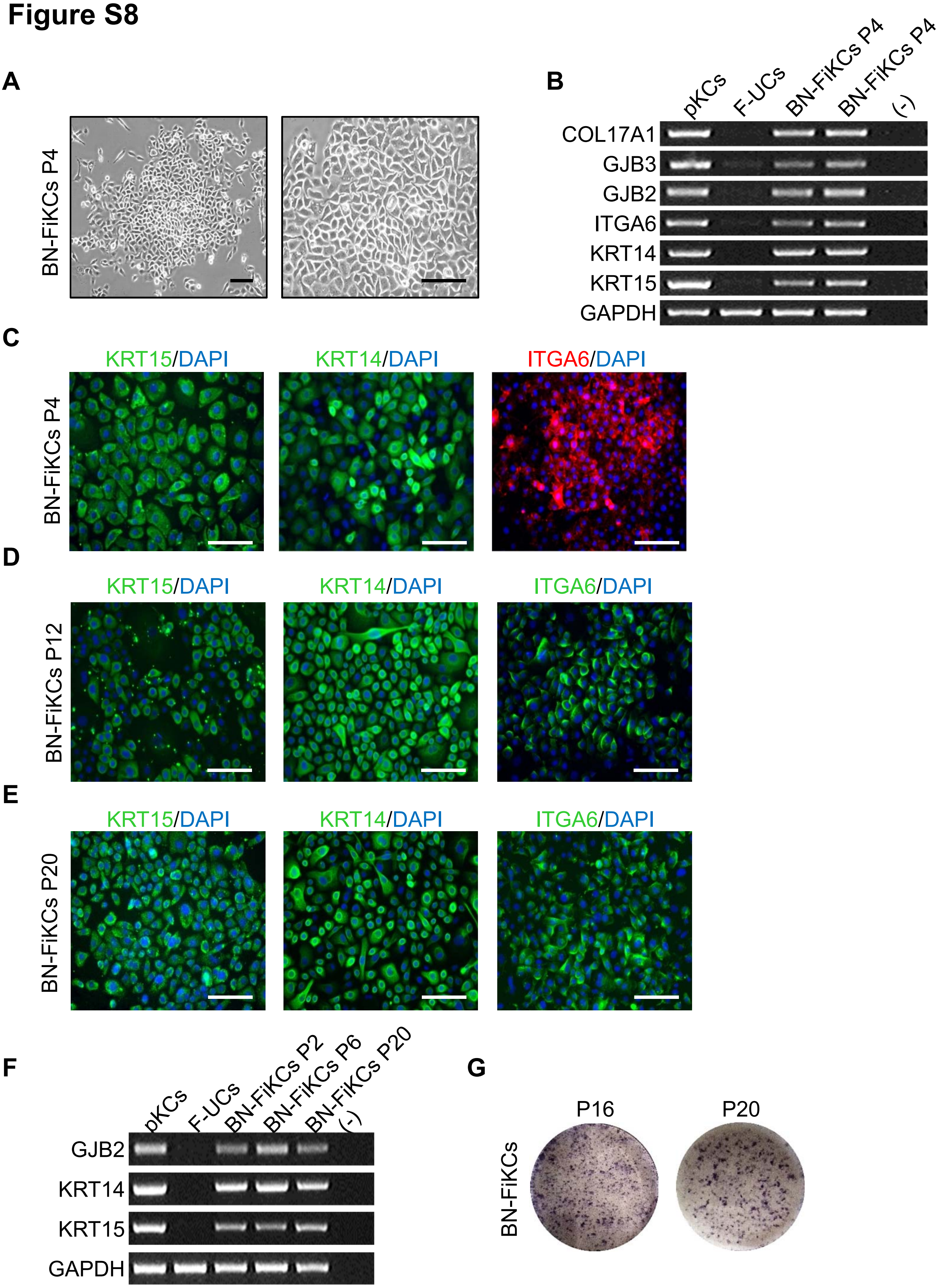
A
BN-FiKCs P4
B
pKCs
F-UCs
BN-FiKCs P4
BN-FiKCs P4
(-)
COL17A1
GJB3
GJB2
ITGA6
KRT14
KRT15
GAPDH
C
KRT15/DAPI
KRT14/DAPI
ITGA6/DAPI
BN-FiKCs P4
D
KRT15/DAPI
KRT14/DAPI
ITGA6/DAPI
BN-FiKCs P12
E
KRT15/DAPI
KRT14/DAPI
ITGA6/DAPI
BN-FiKCs P20
F
pKCs
F-UCs
BN-FiKCs P2
BN-FiKCs P6
BN-FiKCs P20
(-)
GJB2
KRT14
KRT15
GAPDH
G
P16
P20
BN-FiKCs

Figure S9

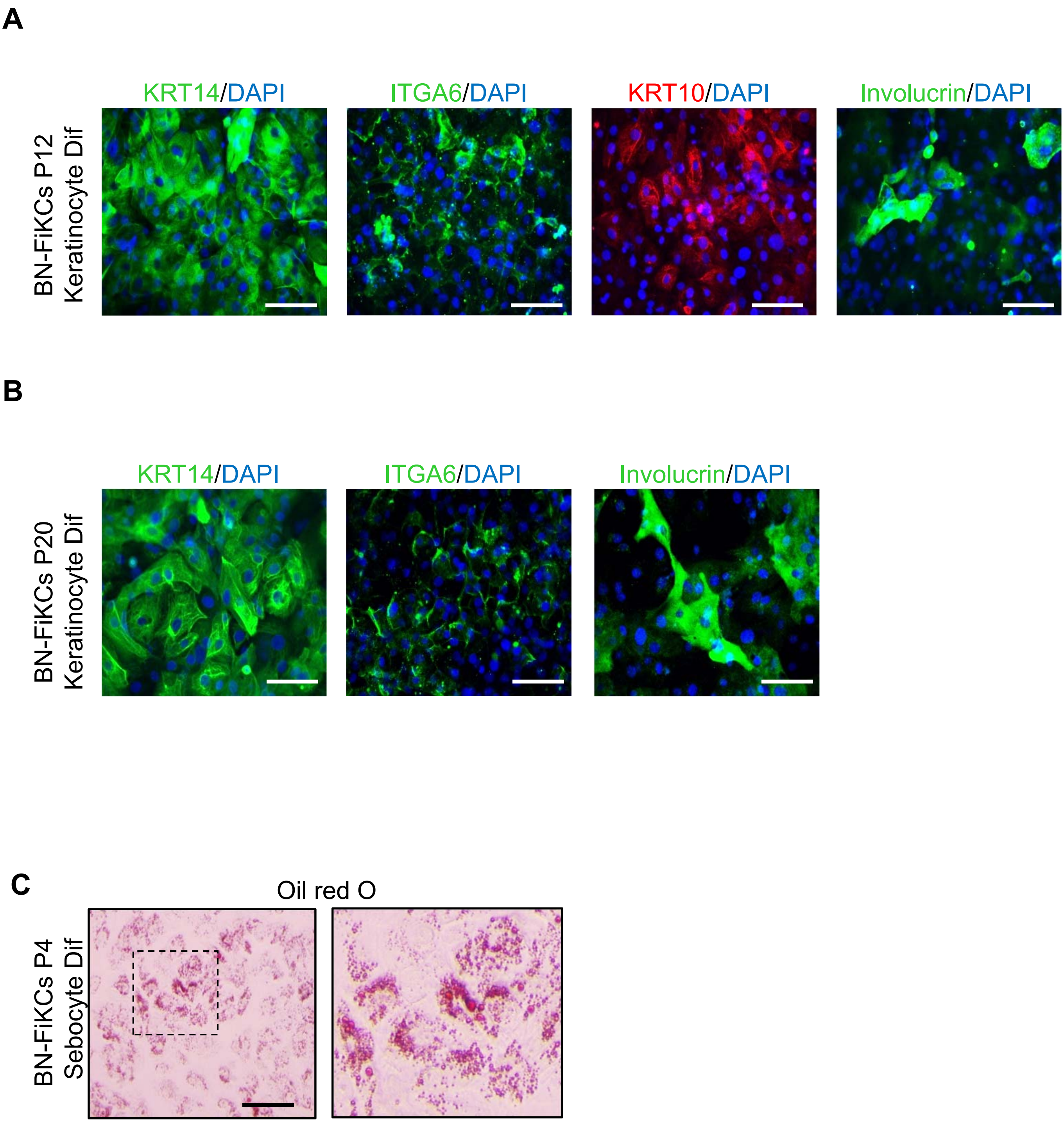
A
KRT14/DAPI
ITGA6/DAPI
KRT10/DAPI
Involucrin/DAPI
BN-FiKCs P12
Keratinocyte Dif
B
KRT14/DAPI
ITGA6/DAPI
Involucrin/DAPI
BN-FiKCs P20
Keratinocyte Dif
C
Oil red O
BN-FiKCs P4
Sebocyte Dif

Figure S10

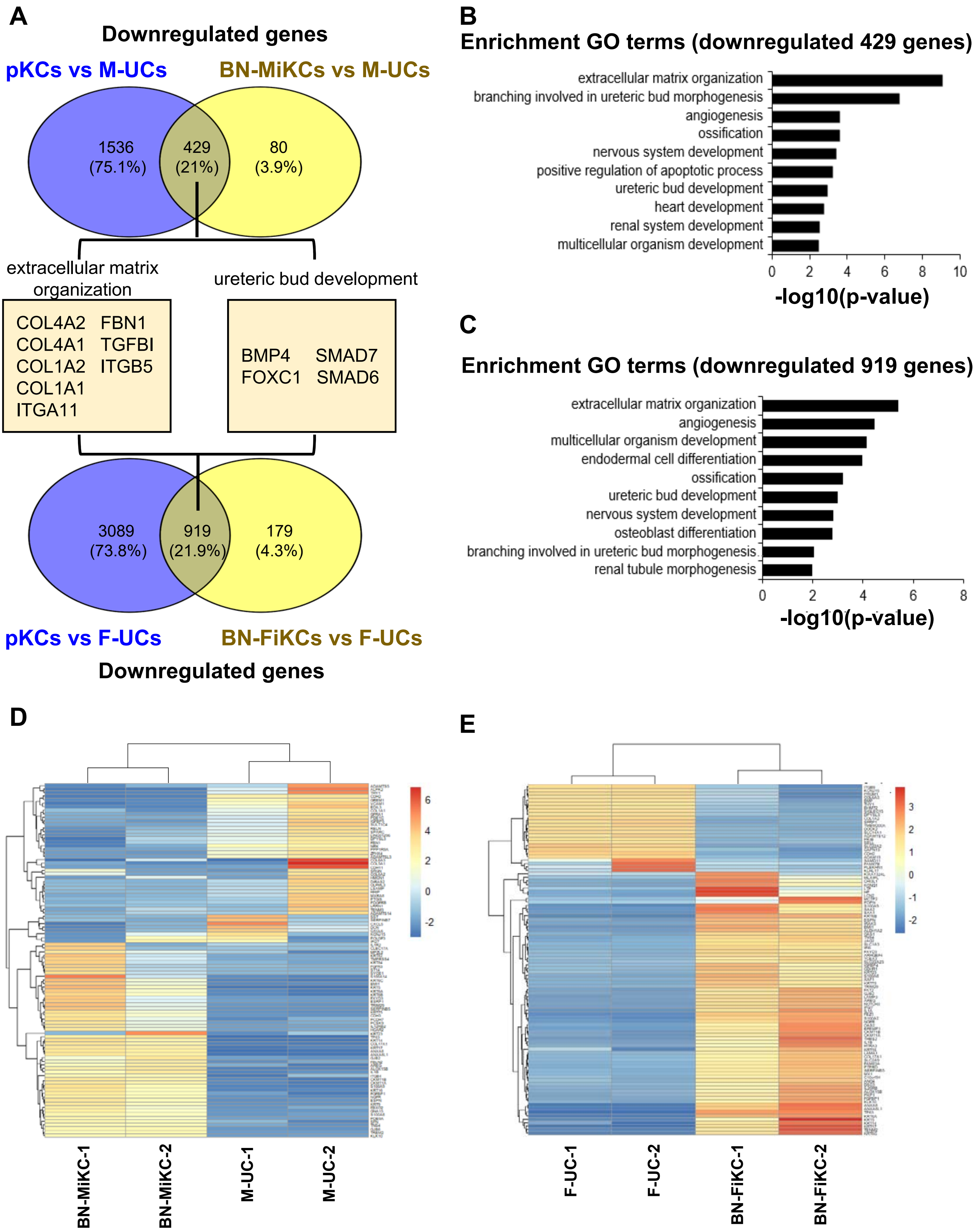

A
Downregulated genes
pKCs vs M-UCs
BN-MiKCs vs M-UCs
1536
(75.1%)
429
(21%)
80
(3.9%)
extracellular matrix organization
ureteric bud development
COL4A2 FBN1
COL4A1 TGFBI
COL1A2 ITGB5
COL1A1
ITGA11
BMP4 SMAD7
FOXC1 SMAD6
3089
(73.8%)
919
(21.9%)
179
(4.3%)
pKCs vs F-UCs
BN-FiKCs vs F-UCs
Downregulated genes
B
Enrichment GO terms (downregulated 429 genes)
extracellular matrix organization
branching involved in ureteric bud morphogenesis
angiogenesis
ossification
nervous system development
positive regulation of apoptotic process
ureteric bud development
heart development
renal system development
multicellular organism development
0 2 4 6 8 10
-log10(p-value)
C
Enrichment GO terms (downregulated 919 genes)
extracellular matrix organization
angiogenesis
multicellular organism development
endodermal cell differentiation
ossification
ureteric bud development
nervous system development
osteoblast differentiation
branching involved in ureteric bud morphogenesis
renal tubule morphogenesis
0 2 4 6 8
-log10(p-value)
D
6 4 2 0 -2
BN-MiKC-1
BN-MiKC-2
M-UC-1
M-UC-2
E
3 2 1 0 -1 -2
F-UC-1
F-UC-2
BN-FiKC-1
BN-FiKC-2

Figure S11

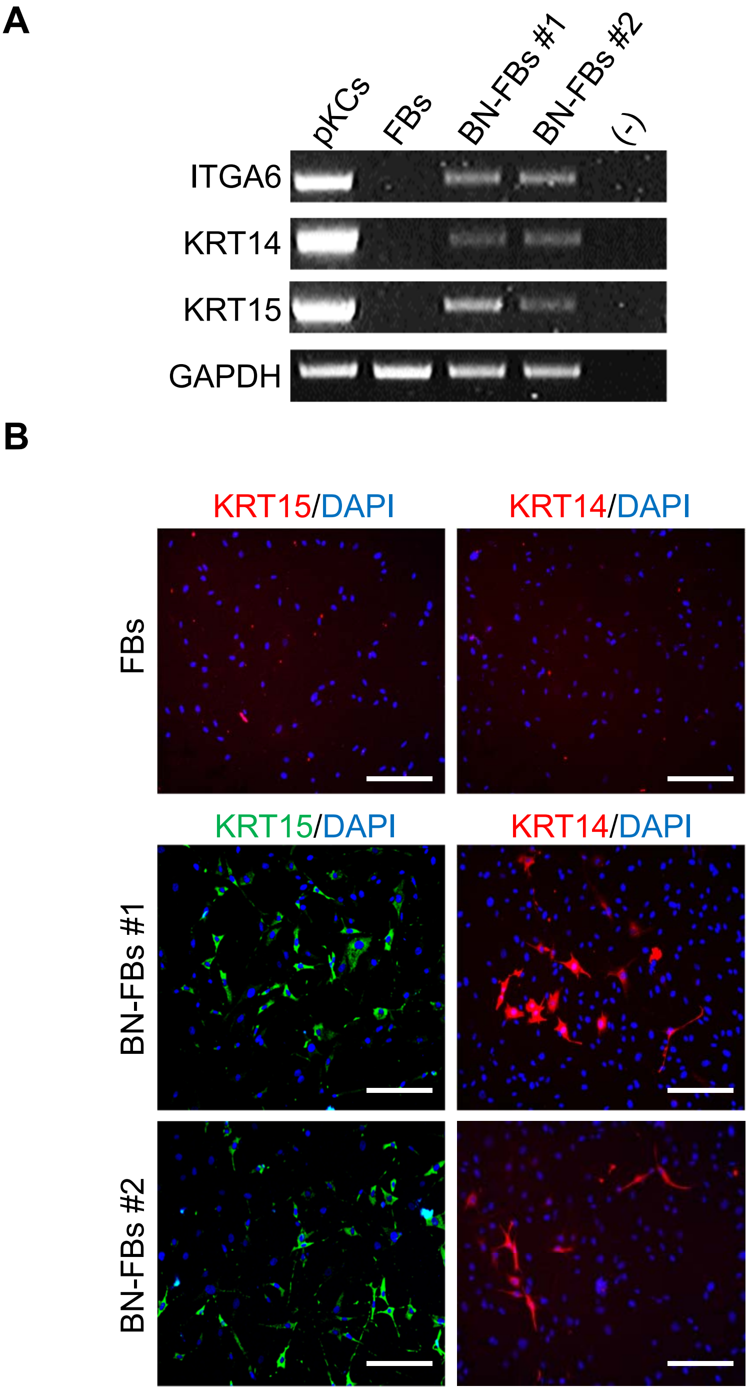

A
pKCs
FBs
BN-FBs #1
BN-FBs #2
(-)
ITGA6
KRT14
KRT15
GAPDH
B
KRT15/DAPI
KRT14/DAPI
FBs
KRT15/DAPI
KRT14/DAPI
BN-FBs #1
BN-FBs #2
